# Italian clinical practice GRADE-based guidelines on the diagnosis and treatment of overweight and obesity, endorsed by the Italian National Institute of Health

**DOI:** 10.1007/s40519-026-01813-z

**Published:** 2026-01-14

**Authors:** Rocco Barazzoni, Silvio Buscemi, Luca Busetto, Paolo Sbraccia, Simona Bo, Emanuele Cereda, Marco Chianelli, Sonja Chiappetta, Riccardo Dalle Grave, Walter de Caro, Giovanni Docimo, Giuseppe Galloro, Primiano Iannone, Frida Leonetti, Fabrizia Lisso, Maria Caterina Manca, Gerardo Medea, Manuela Merli, Anna Maria Moretti, Giuseppe Navarra, Uberto Pagotto, Barbara Paolini, Giovanni Papa, Nicola Perrotta, Andrea Pession, Vincenzo Pilone, Vincenzo Provenzano, Cecilia Ricciardi Rizzo, Maurizio Santomauro, Cristina Segura Garcia, Federico Spandonaro, Samir Sukkar, Patrizia Todisco, Dario Tuccinardi, Andrea Vania, Valentina Vanzi, Riccardo Williams, Iris Zani, Benedetta Ragghianti, Giovanni Antonio Silverii, Amanda Belluzzi, Maria Masulli, Maddalena Redini, Matteo Monami

**Affiliations:** 1 Department of Medical, Surgical and Health Sciences, University of Trieste, Trieste, Italy; 2 Department of Promozione della Salute, Materno-Infantile, Medicina Interna e Specialistica di Eccellenza (PROMISE), University of Palermo, Palermo, Italy; 3 Department of Medicine, University of Padova, Padua, Italy; 4 Department of Systems Medicine, University of Rome Tor Vergata, Rome, Italy; 5 ASO Città della Salute e della Scienza Hospital Department of Medical Sciences, University of Torino, Turin, Italy; 6 SC Dietetica e Nutrizione Clinica Fondazione IRCCS Policlinico San Matteo Pavia, Pavia, Italy; 7 Regina Apostolorum Hospital Albano, Rome, Italy; 8 UOC Chirurgia Generale e Laparoscopica Ospedale Evangelico Betania, Naples, Italy; 9 Department of Eating and Weight Disorders, Villa Garda Hospital, Garda, Italy; 10 Sapienza Università di Roma, Rome, Italy; 11 University of Campania “Luigi Vanvitelli”, Caserta, Italy; 12 Dept. of Clinical Medicine and Surgery Unit of Surgical, Endoscopy University of Naples Federico II - School of Medicine, Naples, Italy; 13 UOC Medicina Interna C, Ospedale Maggiore, Bologna, Italy; 14 Sapienza University of Rome, SM Goretti Hospital, Latin, Italy; 15 Sant’Anna Hospital, Como, Italy; 16 UOS Medicina legale Centro, Azienda USL di Bologna, Bologna, Italy; 17 Medico di Medina Generale ASST Garda, Brescia, Italy; 18 Department of Translation and Precision Medicine - Policlinico Universitario Umberto I- Rome, Rome, Italy; 19 UOC Pneumologia, Università di Bari, Bari, Italy; 20 DU di Patologia Umana DETEV Università di Messina, Messina, Italy; 21 IRCCS Azienda Ospedaliero-Universitaria di Bologna, Bologna, Italy; 22 Dietetica e Nutrizione Clinica, Azienda Ospedaliera Universitaria Senese, Siena, Italy; 23 Head of Plastic Surgery Unit and Plastic Reconstructive and Aesthetic Surgery, University of Trieste, Trieste, Italy; 24 Azienda Ospedaliera Regionale “San Carlo”, Ospedale di Villa d’Agri, Potenza, Italy; 25 Dipartimento di Scienze Mediche e Chirurgiche, Università di Bologna, Bologna, Italy; 26 Dipartimento di Sanità Pubblica, Scuola Medicina e Chirurgia, Università Degli Studi di Napoli “Federico II”, Naples, Italy; 27 Istituto e Clinica S Chiara, Partinico, Palermo, Italy; 28 Department of Public Health, Experimental and Forensic Medicine, University of Pavia, Pavia, Italy; 29 Department of Advanced Biomedical Sciences, University of Naples Federico II, Naples, Italy; 30 Magna Græcia University of Catanzaro, Catanzaro, Italy; 31 Dipartimento di Economia e Istituzioni, University of Rome Tor Vergata, Rome, Italy; 32 U.O. Dietetica e Nutrizione Clinica IRCCS Ospedale Policlinico San Martino di Genova, Genoa, Italy; 33 Psychonutritional Center, Verona, Italy; 34 Fondazione Policlinico Universitario Campus Bio-Medico, Rome, Italy; 35 Independent Researcher, Rome, Italy; 36 Centro Interdipartimentale per la Ricerca e la Formazione, Università degli Studi di Roma Tor Vergata, Rome, Italy; 37 Dipartimento di Psicologia Dinamica e Clinica, Università degli Studi di Roma “Sapienza”, Rome, Italy; 38 Amici Obesi Onlus, Milan, Italy; 39 Diabetology and Metabolic Disease Unit, Careggi Teaching Hospital and University of Florence, Florence, Italy; 40 General Surgery Unit, Ospedale S. Maria Della Misericordia, Rovigo, Italy; 41 University of Naples, Federico II, Naples, Italy; 42 Società Italiana Obesità (SIO), Pisa, Italy

**Keywords:** Obesity, Guidelines, GRADE, Methods

## Abstract

Obesity is a chronic relapsing disease associated with increased morbidity and mortality and reduced quality of life. The present GRADE-based guidelines have been commissioned by the Italian Society of Obesity (SIO) by the Italian National Health Institute to provide evidence-based recommendations on obesity diagnosis and treatment. The panel identified 13 clinical questions, organised into four domains: A. diagnostic criteria (4 questions); B. medical nutrition therapy (4 questions); C. pharmacological, surgical, and endoscopic treatments (4 questions); and D. miscellaneous (1 question). The expert panel recommends adopting at least one anthropometric index of abdominal visceral fat distribution beyond body mass index (BMI) for better clinical risk stratification. A structured lifestyle intervention (i.e., medical–nutritional treatment—MNT), preferably based on cognitive–behavioural therapeutic approach and including physical activity and a balanced diet, should be offered to all subjects living with obesity. Pharmacological or surgical treatment should be offered in addition to MNT when MNT is unable to reach clinical goals. In particular, pharmacological treatment should be considered as the preferred option for subjects with BMI > 27 kg/m^2^ with comorbidities, and for those with BMI 30–39.9 kg/m^2^, based on individual therapeutic goals and needs, adopting surgical treatment in selected cases or in case of OMM failure to reach clinical goals. For subjects with a BMI > 40 kg/m^2^, surgical treatment may be considered conversely as a preferable option. Reduction of at least 10% of the initial body weight is associated with a better quality of life and a reduced risk of incident depression. The current guideline is endorsed by the Italian National Institute of Health, providing the new, updated clinical and legal reference for professionals involved in the management of subjects living with obesity in Italy.

**Supplementary Information:**

The online version contains supplementary material available at 10.1007/s40519-026-01813-z.

## Introduction

Obesity and obesity-related complications (ORCs) represent a growing public health problem in many countries due to their increasing prevalence with significant negative impact on quality of life, and rising economic burden [1]. Treatments for obesity based on lifestyle modification (medical–nutritional therapy—MNT) have limited efficacy, and their impact is often not sustained in the mid-to-long term [2]. Metabolic and bariatric surgery (MBS) has been increasingly introduced to achieve more substantial weight loss with strong superiority in reducing body weight [3–5] and treating conditions, such as Type 2 Diabetes (T2D) [6], arterial hypertension (AH) [7], obstructive sleep apnea syndrome (OSAS) [8], and dyslipidemias (DL) [9]. However, the use of surgical treatment remains limited, due to several barriers, including costs, risks of adverse events, and organizational constraints [10]. Bariatric endoscopy has also been proposed to potentially overcome the above limitations, despite a less pronounced body weight reduction and lower effectiveness on comorbidity remission. Bariatric endoscopy has indeed a better safety profile and higher affordability in terms of direct and indirect costs compared to surgery [11]; however, evidence on the effectiveness profile of bariatric endoscopy is limited with few high-quality studies, relatively small sample size and insufficient follow-up. As a consequence, clinical indications and usefulness of these procedures in treating individuals with overweight and obesity remain incompletely investigated and standardized [11]. Most importantly, pharmacological treatments based on incretin mimetic agents have become available in recent years, potentially combining more substantial weight loss with a safety profile and increasing evidence of a positive impact on several comorbidities.

The development and implementation of rigorous guidelines improves treatment appropriateness with strong potential to enhance the quality and effectiveness of care. In addition, increasing therapeutic options and growing debate on the most appropriate obesity diagnostic frameworks make updated guidance in these fields an urgent priority. The Italian National Institute of Health (ISS) has, therefore, entrusted the Italian Society of Obesity (SIO) to design and develop the first comprehensive Italian guideline aiming at assisting healthcare professionals in selecting the most appropriate diagnostic approach as well as options for the treatment of obesity and its related medical conditions. In the Italian regulatory context, the inclusion of guidelines in the National Guideline System is only possible after a rigorous methodological and formal review by the National Centre for Clinical Excellence under the Ministry of Health. In developing national guidelines, the Centre for Clinical Excellence recommends the use of the GRADE methodology (Grading of Recommendations, Assessment, Development, and Evaluation) [12], which requires the identification of specific clinical questions and the definition of relevant outcomes for each of them. The guideline content is reported in the current paper.

## Methods

This manuscript represents an original scientific work; only the summary of recommendations was non-literally adapted from the Italian SIO guideline document (La diagnosi e la terapia dell’obesità nella popolazione adulta—ISS), while all methodological descriptions, evidence appraisal, and interpretation were independently developed. The detailed methodological process adopted to develop the present Guidelines is reported in a previous publication and Supplementary Materials [13]. Briefly, between June and October 2024, a panel of 39 experts identified by ISS and led by SIO, in collaboration with other scientific societies, as well as an evidence review team (ERT, 4 members), worked to design clinical questions, identify clinical outcomes of interest, and collect and analyze available evidence based on pre-specified search strategies. The panel then provided evidence-based recommendations after a formal vote, using a two-step web-based Delphi method [14]. GRADE methodology and PICO (Patient, Intervention, Comparison, Outcome) conceptual framework [13, 15, 16] have been used to limit the impact of personal opinions and prejudices. Further assessments included economic cost-effectiveness analyses, evaluation of organizational impact, equity, acceptability, and feasibility of recommendations. Healthcare systems, infrastructures, human and financial resources across Italian regions have been considered in its development, and it is, therefore, primarily intended to be applicable in Italy.

The panel identified 13 clinical questions (Table 1), organized into four domains:

A. Diagnostic criteria (4 questions): literature search up to 19/03/2025;
B. Medical nutrition therapy (4 questions): literature search up to 19/03/2025;
C. Pharmacological, surgical, and endoscopic treatments (4 questions): literature search up to 01/12/2024;
D. Miscellaneous (1 question): literature search up to 01/12/2024.

**Table 1:** Summary table of recommendations

N	PICO	*Recommendation*	*Strength of recommendation*	*Quality of evidence*
*Diagnostic criteria*				
1–3	In subjects with BMI between 25 and 34.9 kg/m^2^, is the adoption of anthropometric indexes (i.e., waist circumference—WC, waist-to-hip ratio—WHR, and waist-to-height ratio—WtHR) preferable to BMI alone in the diagnosis and staging of obesity-related complications (ORCs)?	**We recommend, in subjects with BMI between 25 and 34.9 kg/m**^**2**^**, to use at least one anthropometric measurement (i.e., WC, WHR, and WtHR) in addition to BMI to adequately diagnose and stage obesity and its related clinical risk**	*Strong in favor of the intervention*	*Very low*
4	In subjects with BMI between 25 and 34.9 kg/m^2^, is the assessment of body composition, including the use of technical devices and methodologies, preferable to limiting the evaluation to anthropometric indexes, for the staging of obesity-related clinical risk?	**We do not suggest, in subjects with BMI between 25 and 34.9 kg/m**^**2**^**, routine implementation of technical device-based measurements for fat mass quantification with respect to the anthropometric indexes alone, for appropriate staging of obesity-related clinical risk**	*Conditional against the intervention*	*Very low*
*Medical Nutrition Therapy*				
5	In subjects with BMI ≥ 25 kg/m^2^, are structured lifestyle interventions preferable to non-structured lifestyle interventions (LSI) in overweight and obesity management?	**We recommend, in subjects with BMI ≥ 25 kg/m2, structured lifestyle rather than non-structured lifestyle interventions in overweight and obesity management**	*Strong in favor of the intervention*	*Moderate*
6	In subjects with BMI ≥ 25 kg/m^2^ are LSI based on cognitive–behavioral therapy (CBT), preferable to other types of interventions (or standard of care), in overweight and obesity management?	**We recommend, in subjects with BMI ≥ 25 kg/m**^**2**^**, educational interventions based on cognitive–behavioral therapy compared to other types of interventions (i.e., standard of care or no intervention) in overweight and obesity management**	*Strong in favor of the intervention*	*Moderate*
7	We do not recommend in subjects with BMI ≥ 25 kg/m^2^ a ketogenic diet versus balanced macronutrient diets (e.g., Mediterranean diet) for overweight and obesity treatment	**We do not recommend in subjects with BMI ≥ 25 kg/m**^**2**^** a ketogenic diet versus balanced macronutrient diets (e.g., Mediterranean diet) for overweight and obesity treatment**	*Conditional against the intervention*	*Very low*
8	In individuals with BMI ≥ 25 kg/m^2^ are structured LSI, including combined aerobic and anaerobic physical exercise, preferable to structured LSI, including aerobic physical exercise only, for overweight and obesity treatment?	**In subjects with BMI ≥ 25 kg/m**^**2**^**, structured combined lifestyle interventions based on aerobic and anaerobic (combined) exercise are equally effective as those based on aerobic physical exercise only, for overweight and obesity treatment**	*No recommendation*	–
*Pharmacological, surgical, and endoscopic treatments*				
9	In subjects with BMI between 27 and 29.9 kg/m^2^ and ORCs, is the treatment with EMA-approved OMM, in association with MNT, preferable to MNT alone for the treatment and management of overweight?	**We suggest in subjects with a BMI between 27 and 29.9 kg/m**^**2**^** and ORCs, the use of OMM in association with MNT for the treatment of overweight**	*Conditional in favor of the intervention*	*Low quality*
10	In subjects with BMI between 30 and 34.9 kg/m^2^, which anti-obesity strategy (OMM, metabolic bariatric surgery—MBS, or EBP—endoscopic bariatric procedures) associated with MNT, is preferable for the treatment and management of obesity?	**We recommend treating subjects with class I obesity (BMI 30–34, .9 kg/m**^**2**^**), considering OMM as a first-line option in addition to MNT; surgical and endoscopic treatments should be considered as second-line options**	*Strong in favor of the intervention*	*Moderate*
11	In subjects with BMI between 35 and 39.9 kg/m^2^, which anti-obesity strategy (OMM, metabolic bariatric surgery—MBS, or EBP—endoscopic bariatric procedures) associated with MNT, is preferable for the treatment and management of obesity?	**We recommend to treat subjects with class II obesity (BMI 35–39.9 kg/m**^**2**^**) preferring OMM as a first-line option in addition to MNT; surgical and endoscopic treatments are valid options to be preferably adopted after OMM’s failure**	*Strong in favor of the intervention*	*Moderate*
12	In subjects with BMI ≥ 40 kg/m^2^, which anti-obesity strategy (OMM, metabolic bariatric surgery—MBS, or EBP—endoscopic bariatric procedures) associated with MNT, is preferable for the treatment and management of obesity?	**We recommend to treat subjects with class III obesity (BMI ≥ 40 kg/m**^**2**^**) preferring surgical over pharmacological and endoscopic options**	*Strong in favor of the intervention*	*Moderate*
*Miscellaneous*				
13	In subjects with BMI ≥ 30 kg/m^2^, weight loss using either pharmacologic or surgical/endoscopic strategies is preferable to maintaining a stable weight to achieve a psychological well-being?	**We recommend in subjects with BMI ≥ 30 kg/m**^**2**^** a weight loss of at least 5% to achieve (or maintain) a psychological well-being**	*Strong in favor of the intervention*	*Moderate*

The column reported with bold characters corresponds with “recomendations”

The evidence review team identified the characteristics of relevant studies for each critical outcome, defining the search strategy and study inclusion criteria as previously reported [13]. GRADE evaluation for individual critical outcomes has also been performed for each PICO. The guideline applies to adult individuals (age above 18 years) with a body mass index (BMI)≥ 27 kg/m^2^.

## Results

Table 1 reports, in summary, the recommendations for each PICO and the quality of the evidence retrieved.

### Diagnostic criteria

**PICO 1–3:** In subjects with BMI between 25 and 34.9 kg/m^2^, is the adoption of additional anthropometric indexes (i.e., waist circumference—WC, waist-to-hip ratio—WHR, and waist-to-height ratio—WtHR) preferable to BMI alone in diagnosis and staging of obesity and obesity-related complications (ORCs)?

**Table:** 

We recommend, in subjects with BMI between 25 and 34.9 kg/m^2^, to use at least one anthropometric measurement (i.e., WC, WHR, and WtHR) in addition to BMI to adequately diagnose and stage obesity and its related clinical risk	
*Strong recommendation in favor of the intervention, with very low quality of evidence*	

### Rationale

Several studies showed that anthropometric indexes, beyond BMI alone, can be useful in performing an appropriate and accurate diagnosis of obesity [17, 18] and in predicting ORCs (i.e., type 2 diabetes, cardiovascular diseases, cancer), particularly among subgroups of subjects, such as older adults [19] and people with a high percentage of muscle mass [20]. However, available studies provide discordant results [21], which need to be assessed and confirmed by further high-quality literature. For this reason, the ERT decided to perform several analyses and meta-analyses, limiting them to either randomized controlled trials (RCTs) or epidemiological analyses of RCTs. This decision was taken to achieve more rigorous and homogeneous evidence with lower risk of bias. Sensitivity and specificity (diagnostic accuracy) of anthropometric measures and BMI were extracted by available studies and compared with those deriving from dual-energy X-ray absorptiometry (DEXA), and meta-analyses of odds ratios (ORs) for each parameter were performed to assess the predictive values for obesity-related complications.

In the Italian version of the present guidelines PICO 1–3 were split in three different clinical questions (one each for any of the assessed index). We decided to group them together in a single PICO.

The ERT retrieved 7 observational studies [19, 22–27] assessing body fat content with DEXA and 5 epidemiologic analyses of RCTs [21, 28–31] enrolling 26.955 subjects with a mean follow-up of 4.5 years. The analyses performed showed that: (1) the sensitivity of BMI and WC (fewer studies) ranged between 0.30 and 0.90 and 0.80 and 0.99, respectively; (2) BMI was able to adequately predict the risk of developing diabetes and HHF, but not that of MACE and all-cause mortality; (3) WC and WHR were significantly associated with a higher risk of diabetes, HHF, MACE, and mortality; (4) WtHR, although assessed in a smaller number of studies, was significantly associated with a higher risk of MACE and cardiovascular mortality; and (5) visceral fat mass assessed with DEXA has been investigated only for the prediction of incident diabetes, with visceral fat being associated with a statistically significant higher risk for diabetes, but not superior to other indices.

The results of the above-mentioned analyses are extensively reported in Supplementary Materials (Tables S1–S4 and Figs. S1–S5). No pharmaco-economic analyses were retrieved.

### Considerations on specific subgroups of subjects

In selected subgroups, it cannot be excluded that BMI alone can be adequate to stratify the risk of developing ORCs (e.g., subjects with higher degrees of obesity). On the other hand, other subgroups (e.g., athletes with a high percentage of free-fat mass or elderly subjects with sarcopenia) might need more than one additional anthropometric measure, potentially including the assessment of body composition and lean mass with DEXA.

**PICO 4:** In subjects with BMI between 25 and 34.9 kg/m^2^, is assessment of body composition, including use of technical devices and methods, preferable to anthropometric indexes alone, for the staging of obesity-related clinical risk?

**Table:** 

We do not suggest, in subjects with BMI between 25 and 34.9 kg/m^2^, routine implementation of technical device-based measurements for fat mass quantification with respect to the anthropometric indexes alone, for appropriate staging of obesity-related clinical risk	
Conditional recommendation against the intervention, with very low quality of evidence	

### Rationale

Several studies showed that various anthropometric indexes (not limited to BMI alone) may improve the diagnosis of obesity, including better prediction of complications related to excess fat mass (e.g., type 2 diabetes, cardiovascular diseases and cancer), especially in selected subjects’ categories such as older adults [19] and people with higher levels of physical activity [20]. However, a direct evaluation of fat mass using imaging techniques, such as abdominal CT scan or DEXA, might represent an even more useful tool for a more accurate and proper staging of obesity-related clinical risk. To address this clinical question, we took into consideration studies (RCTs and their subgroup analyses) using DEXA or abdominal CT. The ERT retrieved a subgroup analysis from one single RCT (i.e., Diabetes Prevention Program; Tables S5 and S6) [31] showing that total body fat measured by DEXA was associated with an increased risk of diabetes. The accuracy of DEXA was very similar to that obtained for WC.

The results of the above-mentioned analysis are reported in extenso in Supplementary Materials (Tables S5–S7). No pharmaco-economic studies were retrieved; DEXA is associated with higher costs than anthropometric indices measurements, and it is potentially associated with ionizing radiations damages. *Considerations on specific subgroups of subjects.*

Some subgroups of subjects (e.g., older adults, subjects with sarcopenia, etc.) might still benefit from the diagnostic accuracy of DEXA in terms of clinical risk assessment.

### Medical nutrition therapy

**PICO 5:** In subjects with BMI≥ 25 kg/m^2^, are structured lifestyle interventions preferable to non-structured lifestyle interventions (LSI) in overweight and obesity management?

**Table:** 

We recommend, in subjects with BMI ≥ 25 kg/m^2^, structured lifestyle rather than non-structured lifestyle interventions in overweight and obesity management	
Strong recommendation in favor of the intervention, with moderate quality of evidence	

### Rationale

ERT retrieved 18 trials comparing structured and non-structured LSI, showing relevant beneficial effects for structured interventions in terms of fat mass reduction, without any increased risk of adverse events. The lack of long-term follow-up limits the assessment of this intervention. Despite limited evidence about its affordability, other key parameters, including feasibility, applicability, and cost-effectiveness of structured LSI, appear to be superior to non-structured interventions. The results of the above-mentioned analysis are reported in extenso in Supplementary Materials (Figs. S6–S10 and Tables S8 and S12).

### Considerations on specific subgroups of subjects

Some categories of subjects (e.g., those with low literacy levels) might experience greater difficulties in attending and following structured education programs.

**PICO 6:** In subjects with BMI≥ 25 kg/m^2^, are LSI based on cognitive–behavioral therapy (CBT), preferable to other types of interventions (or standard of care), in overweight and obesity management?

**Table:** 

We recommend, in subjects with BMI ≥ 25 kg/m^2^, educational interventions based on cognitive–behavioral therapy compared to other types of interventions (i.e., standard of care or no intervention) in overweight and obesity management	
Strong recommendation in favor of the intervention, with moderate quality of evidence	

### Rationale

ERT retrieved 8 RCTs comparing CBT with other types of structured LSI, showing relevant beneficial effects on fat mass reduction in favor of CBT, without any increased risk of adverse events. The lack of long-term follow-up limits the assessment of this intervention. Despite limited evidence about its affordability, feasibility, applicability, and cost-effectiveness of CBT appears to be superior to other structured interventions. CBT might be recommended wherever required competencies and skills are available. The results of the above-mentioned analysis are reported in extenso in Supplementary Materials (Tables S9 and S12, and Figs. S11–S14).

### Considerations on specific subgroups of subjects

Some categories of subjects (e.g., those with low literacy levels) might experience greater difficulties in attending and following structured education programs.

**PICO 7:** In subjects with BMI≥ 25 kg/m^2^, is a ketogenic diet preferable to balanced macronutrient diets (e.g., Mediterranean diet), for overweight and obesity treatment?

**Table:** 

We do not suggest in subjects with BMI ≥ 25 kg/m^2^ to routinely adopt a ketogenic diet versus balanced macronutrient diets (e.g., Mediterranean diet) for overweight and obesity treatment	
Conditional recommendation against of the intervention, with very low quality of evidence	

### Rationale

The ERT retrieved only 2 head-to-head RCTs, thus limiting the possibility of making a strong recommendation on this clinical question. According to the limited evidence, no clinical advantage of ketogenic diets over balanced macronutrient diets (e.g., Mediterranean diet) was observed. No financial evaluations have been published on this topic so far; however, we can hypothesize a financial/economic disadvantage when pursuing high adherence to the ketogenic diet, based on the need for commercial meal replacements with adequate, high protein and micronutrient intake [32–34]. The results of the above-mentioned analysis are reported in extenso in Supplementary Materials (Table S12 and Figs. S15–S18).

### Considerations on specific subgroups of subjects

A low-calorie ketogenic diet could be useful in selected cases for closely monitored, short-term treatments. On the other hand, subjects affected by eating disorders (e.g., anorexia, bulimia, etc.) could experience a clinical worsening after severe calorie restriction.

**PICO 8:** In subjects with BMI≥ 25 kg/m^2^, are structured LSI, including combined aerobic and anaerobic physical exercise, preferable to structured LSI, including aerobic physical exercise only, for overweight and obesity treatment?

**Table:** 

In subjects with BMI ≥ 25 kg/m^2^, structured combined lifestyle interventions based on aerobic and anaerobic (combined) exercise are equally effective as those based on aerobic physical exercise only, for overweight and obesity treatment	
Conditional recommendation neither in favour nor against the intervention, with low quality of evidence	

### Rationale

ERT retrieved 6 RCTs comparing combined aerobic and anaerobic physical exercise and aerobic physical exercise only. No statistically significant differences have been observed for any of the critical outcomes identified by the panelists. No safety issues were reported. From the financial point of view, no evidence was retrieved. The results of the above-mentioned analysis are reported in *extenso* in Supplementary Materials (Tables S11 and S12; Figs. S19–S21).

For the above reasons, no recommendation was formulated in favor of either type of physical exercise, based on its impact on outcomes regarded as critical. It is of paramount importance to reaffirm that physical exercise programs must be implemented and regarded as fundamental in any structured educational program aimed at treating overweight and obesity.

### Considerations on specific subgroups of subjects

Some categories of subjects might experience difficulty in practicing anaerobic exercise (e.g., older adults) in the absence of a structured intervention supported by dedicated, experienced professionals and specialists. In other cases (e.g., subjects with severe functional limitations), anaerobic exercise might be the only feasible exercise pattern. It is also plausible that anaerobic exercise may have higher clinical relevance and additional benefit for clinical outcomes not regarded as relevant by the ERT. In particular, anaerobic exercise may be more relevant and effective when muscle mass maintenance or recovery is a clinical priority (e.g., in older adults or subjects with sarcopenia).

## Pharmacological, surgical, and endoscopic treatments

**PICO 9:** In subjects with BMI between 27 and 29.9 kg/m^2^ and ORCs, is the treatment with EMA-approved OMM (Obesity Management Medications), in association with MNT, preferable to MNT alone for the treatment and management of overweight?

**Table:** 

We suggest in subjects with a BMI between 27 and 29.9 kg/m^2^ and ORCs, the use of OMM in association with MNT for the treatment of overweight	
Conditional recommendation in favor of the intervention, with low quality of evidence	

### Rationale

Overweight, obesity and ORCs represent rising clinical and economic public health issues due to their dramatic growing prevalence. Although OMMs are indicated for the management of overweight in the presence of ORCs, use of OMMs in subjects with overweight and ORCs is not supported by the same grade of evidence as that available for OMM at higher BMI levels.

The ERT did not retrieve any RCTs specifically performed in subjects with overweight. Two RCTs performed in subjects with BMI> 27 kg/m^2^ reported subgroup analysis on subjects with BMI< 30 kg/m^2^, one each with semaglutide [35] and liraglutide [35], showing significant clinical advantages over placebo. Both liraglutide and semaglutide showed a significantly higher total body weight loss (TBWL)% in comparison with placebo (12.4% and 4.1%, respectively). Semaglutide was associated with a significantly lower incidence of MACE (cardiovascular mortality, stroke and acute coronary ischemia) [36]. Pharmaco-economic studies showed that the treatment of subjects with BMI> 27 kg/m^2^ and ORCs is cost-effective. All the above-mentioned results are reported in extenso in two recent publications [37, 38].

### Considerations on specific subgroups of subjects

The small number of retrieved studies prevented a reliable assessment of the present recommendation in different subgroups of subjects (e.g., those with BMI between 25 and 26.9 kg/m^2^, those with and without ORCs, different age subgroups, women and men, etc.).

**PICO 10:** In subjects with BMI between 30 and 34.9 kg/m^2^, which anti-obesity strategy (OMM, metabolic bariatric surgery—MBS, or EBP—endoscopic bariatric procedures) associated with MNT, is preferable for the treatment and management of obesity?

**Table:** 

We recommend to treat subjects with class I obesity (BMI 30–34, 0.9 kg/m^2^), considering OMM as a first-line option in addition to MNT; surgical and endoscopic treatments should be considered as second-line options	
Strong recommendation in favor of the intervention, with moderate quality of evidence	

### Rationale

Pharmacologic and/or surgical therapy in subjects with obesity, especially when complicated by ORCs, have been assessed in many RCTs, supporting the importance of treating obesity to reduce the risk of mortality and complications.

The ERT retrieved 23 RCTs (24 comparisons) performed in cohorts with BMI between 30 and 34.9 kg/m^2^, comparing several pharmacologic (orlistat, liraglutide, semaglutide, tirzepatide), endoscopic (IGB) and surgical options (gastric bypasses and sleeve gastrectomy) either with placebo or standard of care (SoC). Some treatments showed to be more effective in terms of weight loss (i.e., surgical options, semaglutide and tirzepatide). Among EBP, only endosleeve gastroplasty (ESG) showed a good efficacy profile, despite a high risk of SAE, similar to that observed for MBS. Semaglutide was associated with a significant reduction of all-cause mortality and risk of incident MACE, type 2 diabetes, and a higher chance of diabetes remission.

Pharmaco-economic studies showed that both surgical and pharmacologic options are cost-effective. Notably, cost of OMM is not currently reimbursed in Italy, while cost of MBS and EBP is, rising possible equity concerns. All the mentioned-above results are reported in extenso in two recent publications [37, 38].

### Considerations on specific subgroup of subjects

The relatively small number of pre-planned subgroup analyses of retrieved studies prevented a reliable assessment of the present recommendation in different subgroups of subjects (e.g., those with and without ORCs, different age subgroups, women and men, etc.)

**PICO 11:** In subjects with BMI between 35 and 39.9 kg/m^2^, which anti-obesity strategy (OMM, metabolic bariatric surgery—MBS, or EBP—endoscopic bariatric procedures) associated with MNT, is preferable for the treatment and management of obesity?

**Table:** 

We recommend to treat subjects with class II obesity (BMI 35–39.9 kg/m^2^) preferring OMM as a first-line option in addition to MNT; surgical and endoscopic treatments are valid options to be preferably adopted in case of failure of OMM	
Strong recommendation in favor of the intervention, with moderate quality of evidence	

### Rationale

Pharmacologic and/or surgical therapy in subjects with obesity, especially when complicated by ORCs, have been assessed in many RCTs, supporting the importance of treating obesity to reduce the risk of mortality and complications.

The ERT retrieved 57 RCTs performed in subjects with BMI between 35 and 39.9 kg/m^2^, comparing different pharmacologic, endoscopic and surgical therapeutic strategies either with placebo or SoC. Some therapeutic strategies were associated with higher TBWL%, including most surgical procedures (except for laparoscopic adjustable gastric banding—LAGB), tirzepatide, semaglutide, and ESG. Surgical and endoscopic treatments are associated with a higher risk of serious adverse events, while no relevant safety issues were reported for OMM, except NB.

All OMMs, except orlistat, as well as RYGB and SG, effectively increase diabetes remission rate. Semaglutide and tirzepatide were associated with a lower risk of hospitalization for heart failure, and tirzepatide with a higher rate of OSAS and MASH (Metabolic-Associated Steato-Hepatitis) remission, and an improvement of hepatic fibrosis.

Pharmaco-economic studies show that both surgical and pharmacologic therapies have optimal cost-effectiveness profiles. Notably, the cost of OMM is not currently reimbursed in Italy, while the cost of MBS and EBP is, raising possible equity concerns. All the above-mentioned results are reported in extenso in two recent publications [37, 38].

### Considerations on specific subgroups of subjects

The relatively small number of pre-planned subgroup analyses of retrieved studies prevented reliable assessment of the present recommendation in different subgroups of subjects (e.g., those with and without ORCs, different age subgroups, women and men, etc.).

**PICO 12:** In subjects with BMI ≥ 40kg/m^2^, which anti-obesity strategy (OMM, metabolic bariatric surgery—MBS, or EBP—endoscopic bariatric procedures) associated with MNT, is preferable for the treatment and management of obesity?

**Table:** 

We recommend to treat subjects with class III obesity (BMI ≥ 40 kg/m^2^) preferring surgical over pharmacological and endoscopic options, in addition to MNT	
Strong recommendation in favor of the intervention, with moderate quality of evidence	

### Rationale

Severe obesity and its ORCs also represent a rising public health issue due to a growing prevalence, a relevant impact on subjects’ health and quality of life and a huge economic burden-related. Pharmacologic and/or surgical therapy in subjects with severe obesity, especially when complicated by ORCs, are supported by several lines of evidence.

The ERT retrieved 54 RCTs, all performed with MBS, with the exception of 3 studies, one and two RCTs with semaglutide and intragastric balloon (IGB), respectively. MBS (RYGB, OAGB, LVGB, GCP, SG, SADI e BPD) effectively reduced body weight in the long term (with the exception of LAGB and GCP), with an increased risk of SAE, particularly for BPD and SAD, and LVGB. Among OMMs, only semaglutide has been assessed in this class of obesity reporting a TBWL of 10% in the short term; this effect was statistically inferior to those observed for MBS (>15–20%). No RCTs were retrieved for EBP.

Pharmaco-economic studies show that all treatment strategies are cost-effective. Cost of OMM is not currently reimbursed in Italy raising possible equity concerns, since the cost of MBS and EBP is reimbursed. All the above-mentioned results are reported in extenso in two recent publications [37, 38].

### Considerations on specific subgroups of subjects

Subjects at high risk for surgical complications (e.g., BMI>50 kg/m^2^, multiple comorbid conditions, etc.) or with the highest BMI might benefit from OMMs pre- and/or post-surgical treatment. Some OMMs (e.g., tirzepatide) have not been extensively studied in subjects with BMI≥ 40 kg/m^2^, but their efficacy has been widely proven, and their use could be useful in this class of obesity.

## Miscellaneous

**PICO 13:** In subjects with BMI≥ 30 kg/m^2^, is weight loss using either pharmacological or surgical/endoscopic strategies preferable to maintaining a stable weight to achieve a psychological well-being?

**Table:** 

We recommend in subjects with BMI ≥ 30 kg/m^2^ a weight loss of at least 5% to achieve (or maintain) a psychological well-being	
Strong recommendation in favor of the intervention, with moderate quality of evidence	

### Rationale

It is widely acknowledged that excess body weight is strongly linked with social stigma, potentially affecting the psychological well-being of subjects living with obesity [39]. Weight loss should theoretically improve the quality of life and reduce the risk of incident depression and anxiety, as suggested by several studies [40]. Recently, some concerns have been raised about psychiatric safety for some OMMs [39, 40]. Relevant weight loss following surgical procedures [41] or new incretin-mimetic OMMs might unmask hidden, pre-existing psycho-pathological alterations [42].

The ERT retrieved 26 RCTs determining a placebo-subtracted TBWL of at least 5%, showing that: (1) weight loss greater than 5% produces a 28% risk reduction of incident depression, irrespective of mean entry BMI. A greater weight loss (at least 10%) seems to be associated with a lower risk of incident depression; (2) weight loss is not associated with any change in the incidence of anxiety; and (3) weight loss, irrespective of the implemented strategy (surgical or pharmacological), is associated with a general improvement of self-reported quality of life.

No pharmaco-economic studies were retrieved on this topic.

The cost of OMM is not currently reimbursed in Italy, rising possible equity concerns, since the cost of MBS and EBP is reimbursed. All the above-mentioned results are reported in extenso in a recent publication [37, 38, 43].

### Considerations on specific subgroups of subjects

We cannot exclude that in some subgroups (e.g., elderly, subjects with previous or actual psychiatric disorders) weight loss might be associated with different outcomes from those reported in the present recommendation.

## Discussion

The growing burden of obesity and the large amount of published clinical trials exploring newer pharmacological and surgical therapies create an urgent priority to update recommendations on obesity treatment. Moreover, several concerns have been raised about the diagnostic criteria of obesity and the limitations related to the use of BMI alone to define a broad spectrum of chronic and multifactorial organ dysfunctions [43–45]. Indeed, considering the current legislation on professional liability, correct, appropriate indications for diagnosis and treatment of obesity can support clinicians in the context of increasing legal claims. In this context, the Italian Society of Obesity has been invited by the Italian National Institute of Health (ISS) to develop a GRADE-based guideline on the diagnosis and treatment of obesity. The broader areas covered by clinical questions included in the present guidelines were: diagnostic criteria, MNT, pharmacological, surgical, and endoscopic therapies, and psychological impact of weight loss.

The first challenge in facing obesity and its related complications is represented by diagnostic criteria, which are increasingly debated by scientific societies and consensus groups, without a full agreement on tools and thresholds to adopt. The majority of authors and scientific societies agree that BMI alone is inadequate for optimal identification of excess fat mass and the risk of incident ORCs, proposing different solutions and tools to overcome BMI limitations. For instance, the Lancet Commission recently proposed the use of at least one anthropometric index in addition to BMI [17]; EASO suggests the use of the waist-to-height ratio [44], etc.). However, no formal analyses to assess the superiority of an approach over the other have been attempted so far. We, therefore, collected studies comparing anthropometric indices with BMI thresholds, and found that concomitant use of waist circumference (or its related indices) increases the ability of identifying fat mass excess (using DEXA as the reference category) and subsequent risk of ORCs. The routine use of waist circumference-based anthropometric indices can, therefore, provide useful information, not only on the amount of body fat excess, but also about its distribution, which is independently correlated with the risk of cardiovascular and metabolic disease. Use of such indices is, therefore, recommended in addition to BMI. Although the certainty of evidence supporting this recommendation was rated as very low, the panel issued a strong recommendation based on GRADE Evidence-to-Decision considerations, including the absence of harm, minimal resource use, high feasibility, wide acceptability, and the potential for clinically meaningful improvement in cardiometabolic risk stratification. This approach was deemed proportionate and aligned with patient and clinician values, despite limitations in the available evidence.

Another pivotal topic addressed by the present guideline is the importance of medical nutrition therapy. Based on retrieved evidence and meta-analyses, we confirmed the efficacy of structured LSI, particularly those based on cognitive–behavioral approaches, compared to the standard of care (dietary or physical activity advice). A structured intervention, in fact, is aimed at providing knowledge and skills required for implementing a healthy lifestyle, promoting a holistic approach, taking into consideration also psychological and emotional aspects. Structured education programs can facilitate a therapeutic alliance between healthcare professionals and subjects living with obesity as well as their caregivers, thereby increasing therapeutic adherence and compliance. Educational programs based on CBT appear to be more effective than other types of interventions, although their adoption requires additional skills and organization models that may be cumbersome to implement in all centers involved in the management of obesity. The conditional recommendation against ketogenic diets was primarily based on the lack of demonstrated clinical superiority over balanced dietary approaches and concerns regarding long-term adherence, while economic considerations were regarded as secondary and hypothesis-generating in the absence of formal cost-effectiveness analyses.

Another important aspect addressed by the present guideline is the assessment of available pharmacological and surgical treatments and their role in obesity management. Similar to other therapeutic algorithms [45–47], the present guideline assessed the efficacy and safety of different anti-obesity strategies. The novelty of the present work is that of providing stratified recommendations for different classes of obesity. Based on available evidence, subjects affected by mild to moderate obesity (class I and II) should be treated preferentially with OMMs added to MNT. Among subjects with higher BMI (class III of obesity), the surgical option should be considered as the first-line option whenever possible. The use of multimodal strategies with MNT associated with OMMs and MBS could be most effective in several selected cases, after careful assessment of efficacy, safety, and costs. The current recommendations also specifically took into account the different efficacy and safety profiles for OMMs and MBS, and other parameters, including but not limited to effects on ORCs treatment and prevention, cost-effectiveness, etc. MBS has been traditionally considered more effective than OMMs despite a higher risk of SAE. Newer incretin-mimetic OMMs have, however, become available (i.e., semaglutide and tirzepatide) with greater efficacy than previously available agents (i.e., orlistat, naltrexone–bupropion, and liraglutide). When comparing individual anti-obesity strategies in trials enrolling subjects with lower classes of obesity (i.e., Classes I and II), newer OMMs seem to be competitive towards certain surgical procedures (i.e., SG, LAGB, and GCP) and not inferior to gastric bypass and SADI or BPD [37, 38, 43]. On the other hand, the safety profile of OMMs remains considerably better than any surgical intervention. Based on these findings, the expert panel decided to recommend the use of newer OMMs as first-line therapies in class I and II obesity, considering MBS only in case of treatment failure or insufficient weight loss. In the highest obesity class with BMI>40 kg/m^2^, MBS (in particular, gastric bypass, SG, SADI, and BPD) still represents the first option due to its greater efficacy in terms of weight loss and the very limited evidence on OMMs. Several types of MBS seem to be preferable over others; RYGB, OAGB, and SG are more effective (i.e., LAGB and GCP) or have better safety profile (i.e., SADI and BPD) among metabolic and bariatric surgical interventions [37, 38, 43]. From a feasibility and equity perspective, the panel acknowledged that OMM are not currently reimbursed within the Italian National Health Service, which may represent a relevant barrier to implementation. However, this recommendation reflects clinical appropriateness and proportionality based on available evidence, and highlights a potential misalignment between evidence-based care and current reimbursement policies, with implications for equity that warrant consideration in future health policy decisions.

The last PICO of the present guidelines addressed the effects of weight loss on quality of life and the psychological domain. Available evidence has shown that treatments inducing weight loss are not associated with detrimental effects on mental health; conversely, weight loss seems to be associated with a reduced risk of depression, particularly for weight loss greater than 10%. The only relevant exception is the combination of naltrexone/bupropion, which was reportedly associated with an increased risk of anxiety [43].

## Limitations

Several limitations should be acknowledged, particularly for PICOs regarding pharmacological and surgical treatments of obesity. The quality of trials is not homogeneous, possibly introducing several biases. The open-label design inevitably adopted in many of the included RCTs on MBS could have introduced a bias in favor of surgical approaches in comparison with OMMs, which are usually compared to placebo (the placebo effect is not null in terms of weight loss). Moreover, to compare different surgical and nonsurgical strategies for treating obesity, the ERT was forced to perform network meta-analyses with a heterogeneous reference category (i.e., placebo/lifestyle interventions/standard of care). In fact, most RCTs performed on OMMs are usually placebo-controlled, whereas EBP and MBS are often compared to lifestyle interventions or no therapy (i.e., uncontrolled studies). This could represent a source of heterogeneity, and it could have introduced a bias against OMMs, underestimating the actual impact on the primary endpoint. Moreover, limited information on the long-term efficacy of pharmacological treatments and different follow-ups and treatment across different anti-obesity strategies makes formal comparisons between OMMs, EBP, and MBS more difficult. Finally, despite favorable cost-effectiveness profiles of all anti-obesity strategies analyzed, a formal comparison across different strategies is complex due to different reimbursement policies in Italy. MBS and EBP, but not OMMs, are reimbursed from the Italian National Health Institute, thus generating potential inequity based on socioeconomic status, as previously stated.

## Conclusions

In conclusion, the present guidelines, endorsed by the Italian National Institute of Health, represent the new clinical and legal reference for all professionals involved in the management of subjects living with obesity in Italy. The main recommendations included in these guidelines refer to diagnostic and therapeutic clinical questions. In summary, the expert panel strongly recommend, as suggested by available literature, adopting at least one anthropometric index, beyond BMI, to better diagnose and stage obesity. Moreover, a structured lifestyle intervention (i.e., MNT; preferably based on cognitive–behavioral therapy and including physical activity and a balanced macronutrient diet) should be offered to all subjects living with obesity. The use of pharmacological and surgical treatments, in addition to MNT, should be based on therapeutic goals and needs, considering OMM (particularly tirzepatide and semaglutide) as a first-line option, except for cases with the highest obesity class or in cases of OMM failure to reach individualized clinical goals.

## Supplementary Information

Below is the link to the electronic supplementary material.

Supplementary file 1.

## Data Availability

No datasets were generated or analysed during the current study.
