# Supplementary material for: Italian clinical practice GRADE-based guidelines on the diagnosis and treatment of overweight and obesity, endorsed by the Italian National Institute of Health
Source: Eat Weight Disord. 2026 Jan 14;31(1):9. doi: 10.1007/s40519-026-01813-z (PMC12880997; doi:10.1007/s40519-026-01813-z)
Supplement: Supplementary file 1 [file 40519_2026_1813_MOESM1_ESM.docx]

**SUPPLEMENTARY MATERIALS**

***1. DIAGNOSTIC CRITERIA***

**Questions**

**PICO 1**

*Pubmed: # 4072*

*Search: obesity and (BMI or waist or "body fat" or "body composition" or "lean mass") and (diabetes or cardiovascular or cancer or mortality or "sleep apnea" or steato-hepatitis) Filters: Randomized Controlled Trial*

*(("obeses"[All Fields] OR "obesity"[MeSH Terms] OR "obesity"[All Fields] OR "obese"[All Fields] OR "obesities"[All Fields] OR "obesity s"[All Fields]) AND ("BMI"[All Fields] OR ("waist"[All Fields] OR "waists"[All Fields]) OR "body fat"[All Fields] OR "body composition"[All Fields] OR "lean mass"[All Fields]) AND ("diabete"[All Fields] OR "diabetes mellitus"[MeSH Terms] OR ("diabetes"[All Fields] AND "mellitus"[All Fields]) OR "diabetes mellitus"[All Fields] OR "diabetes"[All Fields] OR "diabetes insipidus"[MeSH Terms] OR ("diabetes"[All Fields] AND "insipidus"[All Fields]) OR "diabetes insipidus"[All Fields] OR "diabetic"[All Fields] OR "diabetics"[All Fields] OR "diabets"[All Fields] OR ("cardiovascular system"[MeSH Terms] OR ("cardiovascular"[All Fields] AND "system"[All Fields]) OR "cardiovascular system"[All Fields] OR "cardiovascular"[All Fields] OR "cardiovasculars"[All Fields]) OR ("cancer s"[All Fields] OR "cancerated"[All Fields] OR "canceration"[All Fields] OR "cancerization"[All Fields] OR "cancerized"[All Fields] OR "cancerous"[All Fields] OR "neoplasms"[MeSH Terms] OR "neoplasms"[All Fields] OR "cancer"[All Fields] OR "cancers"[All Fields]) OR ("mortality"[MeSH Terms] OR "mortality"[All Fields] OR "mortalities"[All Fields] OR "mortality"[MeSH Subheading]) OR "sleep apnea"[All Fields] OR "steato-hepatitis"[All Fields])) AND (randomizedcontrolledtrial[Filter])*

*Translations*

*obesity: "obeses"[All Fields] OR "obesity"[MeSH Terms] OR "obesity"[All Fields] OR "obese"[All Fields] OR "obesities"[All Fields] OR "obesity's"[All Fields]*

*waist: "waist"[All Fields] OR "waists"[All Fields]*

*diabetes: "diabete"[All Fields] OR "diabetes mellitus"[MeSH Terms] OR ("diabetes"[All Fields] AND "mellitus"[All Fields]) OR "diabetes mellitus"[All Fields] OR "diabetes"[All Fields] OR "diabetes insipidus"[MeSH Terms] OR ("diabetes"[All Fields] AND "insipidus"[All Fields]) OR "diabetes insipidus"[All Fields] OR "diabetic"[All Fields] OR "diabetics"[All Fields] OR "diabets"[All Fields]*

*cardiovascular: "cardiovascular system"[MeSH Terms] OR ("cardiovascular"[All Fields] AND "system"[All Fields]) OR "cardiovascular system"[All Fields] OR "cardiovascular"[All Fields] OR "cardiovasculars"[All Fields]*

*cancer: "cancer's"[All Fields] OR "cancerated"[All Fields] OR "canceration"[All Fields] OR "cancerization"[All Fields] OR "cancerized"[All Fields] OR "cancerous"[All Fields] OR "neoplasms"[MeSH Terms] OR "neoplasms"[All Fields] OR "cancer"[All Fields] OR "cancers"[All Fields]*

*mortality: "mortality"[MeSH Terms] OR "mortality"[All Fields] OR "mortalities"[All Fields] OR "mortality"[Subheading]*

Tables

**Table 1** – Accuracy of different anthropometric indexes (BMI≥ 30 kg/m^2^; waist circumference ≥88/102 cm in men and women respectively, WTH*≥*0.95/0.80 in men and women respectively; WtHR≥ 0.50) in obesity diagnosis (DEXA: fat mass *≥* 25/35% in men and women respectively).

| *Name* (number of subjects) | *Categories* | ***BMI*** | | | | | | | ***Waist circumference*** | | | | | | |
| --- | --- | --- | --- | --- | --- | --- | --- | --- | --- | --- | --- | --- | --- | --- | --- |
|  |  | ***Sens.*** | ***Spec.*** | *PPV* | *NPV* | ***CC*** | *FP* | *FN* | *Sens.* | *Spec.* | *PPV* | *NPV* | *CC* | *FP* | *FN* |
| *Ha**yman^8^*^§^  *(1,394)* | Total | 0.74 | 0.65 | 0.74 | 0.65 | 70.2 | 15.1 | 14.7 | *-* | *-* | *-* | *-* | *-* | *-* | *-* |
|  | *Women* | *0.77* | 0.76 | 0.83 | 0.74 | *76.4* | *10.1* | 13.5 | *-* | *-* | *-* | *-* | *-* | *-* | *-* |
|  | *Men* | *0.73* | 0.62 | 0.71 | 0.64 | *68.0* | *16.9* | 15.1 | *-* | *-* | *-* | *-* | *-* | *-* | *-* |
| *Alammar^9^*^§^  *(942)* | Total | - | - | - | - | - | - | - | *-* | *-* | *-* | *-* | *-* | *-* | *-* |
|  | *Women* | *0.55* | 0.93 | - | - | *-* | *-* | - | *-* | *-* | *-* | *-* | *-* | *-* | *-* |
|  | *Men* | *0.34* | 0.98 | - | - | *-* | *-* | - | *-* | *-* | *-* | *-* | *-* | *-* | *-* |
| *Alqarn^10^*^§^  *(319)** | Total | - | - | - | - | - | - | - | *-* | *-* | *-* | *-* | *-* | *-* | *-* |
|  | *Women* | *0.57* | 0.88 | 0.99 | 0.12 | *58.6* | *-* | - | *-* | *-* | *-* | *-* | *-* | *-* | *-* |
|  | *Men* | *0.48* | 1.00 | 1.00 | 0.23 | *55.2* | *-* | - | *-* | *-* | *-* | *-* | *-* | *-* | *-* |
| *Batsis^13^*^§^  *(4,984)*** | Total | - | - | - | - | - | *-* | - | - | - | - | - | - | *-* | - |
|  | *Women* | *0.38* | 0.98 | 0.99 | 0.88 | *45.1* | *-* | - | 0.80 | 0.85 | 0.98 | 0.66 | *81.0* | *-* | *-* |
|  | *Men* | *0.33* | 0.99 | 0.99 | 0.17 | *41.0* | *-* | - | 0.60 | 0.95 | 0.99 | 0.74 | *64.2* | *-* | *-* |
| Porto^12§^  *(3,822)* | Total | 0.47 | 0.93 | 0.57 | 0.90 | 85.8 | 5.8 | 8.3 | *-* | *-* | *-* | *-* | *-* | *-* | *-* |
|  | *Women* | *-* | - | - | - | *-* | *-* | - | *-* | *-* | *-* | *-* | *-* | *-* | *-* |
|  | *Men* | - | - | - | - | - | - | - | *-* | *-* | *-* | *-* | *-* | *-* | *-* |
| Vasconcelos^5§^  *(180)* | Total |  |  |  |  |  |  |  | *-* | *-* | *-* | *-* | *-* | *-* | *-* |
|  | *Women* | *0.29* | 1.00 | - | - | *32.5* | *0.0* | 67.5 | *-* | *-* | *-* | *-* | *-* | *-* | *-* |
|  | *Men* | *0.32* | 0.97 | - | - | *66.6* | *1.7* | 21.7 | *-* | *-* | *-* | *-* | *-* | *-* | *-* |
| Zwierzchowska^11§^  *(87)* | Total | *-* | - | - | - | *-* | *-* | - | *-* | - | - | - | *-* | *-* | - |
|  | *Women* | *0.90* | 0.74 | - | - | *-* | *-* | - | 0.99 | 0.58 | - | - | *-* | *-* | *-* |
|  | *Men* | *-* | - | - | - | *-* | *-* | - | *-* | - | - | - | *-* | *-* | - |
| *Name*  (# pts) | *Categories* | **BMI** | | | | | | | ***WtHR*** | | | | | | |
| Zwierzchowska^11§^  *(87)* | Total | *-* | - | - | - | *-* | *-* | - | *-* | - | - | - | *-* | *-* | - |
|  | *Women* | *0.90* | 0.74 | - | - | *-* | *-* | - | 1.00 | 0.84 | - | - | *-* | *-* | *-* |
|  | *Men* | *-* | - | - | - | *-* | *-* | - | *-* | - | - | - | *-* | *-* | - |

Sens.: Sensibility; Spec.: Specificity; PPV: positive predictive value; NPV: negative predictive value; CC: Correctly classified: %; FP: False positives; FN: False negatives. WTH: waist to hip ratio; WtHR: Waist to height ratio.

**Table 2** – Predictive value of single anthropometric indexes for different obesity – related comorbidities. Relative risk for every incremental point of single parameters. Analysis performed on epidemiological analysis of clinical trials.

| **First author** | **n. subjects** | **BMI**  (kg/m^2^) | | **Waist circumference** (cm) | | **Waist to hip ratio** | | **Waist to height ratio** | | **Fat mass**  (%) | |
| --- | --- | --- | --- | --- | --- | --- | --- | --- | --- | --- | --- |
|  |  | OR/AUC | *95%, CI* | OR/AUC | *95%, CI* | OR/AUC | *95%, CI* | OR/AUC | *95%, CI* | OR/AUC | *95%, CI* |
| **Diabetes** | | | | | | | | | | | |
| Bray^17^  Men  Women | Placebo  108 | **1.41**  0.98 | [1.10;4.82]  [0.71;1.35] | **1.69**  1.02 | [1.14;2.51]  [0.75;1.40] | **1.44**  1.31 | [1.00;2.07]  [0.98;1.75] | **1.49**  0.98 | [1.07;2.07]  [0.71;1.35] | **1.48****  1.28 | [1.02;2.14]  [0.96;1.70] |
| Bray^17^  Men  Women | Metformin  112 | 1.56  0.95 | [0.86;2.82]  [0.66;1.39] | 1.49  1.03 | [0.79;2.84]  [0.71;1.49] | 1.38  1.17 | [0.87;2.19]  [0.83;1.65] | 1.66  1.00 | [0.90;3.05]  [0.72;1.38] | 1.67**  1.16 | [0.95;2.93]  [0.82;1.64] |
| Bray^17^  Men  Women | Lifestyle  111 | 1.37  **1.57** | [0.60;3.14]  [1.02;2.43] | 1.50  1.43 | [0.69;3.25]  [0.89;2.29] | **3.47**  1.31 | [1.36;8.89]  [0.89;1.93] | 1.65  **1.57** | [0.78;3.48]  [1.02;2.43] | 1.61**  1.40 | [0.82;3.15]  [0.92;2.14] |
| **Cardiovascular events (MACE)** | | | | | | | | | | | |
| Franek^14^ | 4,592* | 1.06 | [0.98;1.15] | **1.12** | [1.02;1.22] | **1.11** | [1.05;1.21] | - | - | - | - |
| German^7^ | 10,251 | 0.97 | [0.91;1.03] | **1.07** | [1.01;1.13] | - | - | - | - | - | - |
| Rådholm^16^ | 11,125 | **1.09** | [1.04;1.14] | **1.15** | [1.09;1.21] | **1.09** | [1.04;1.15] | **1.16** | [1.11;1.22] | - | - |
| Kadakia^15^ | 6,560 | 1.09 | [0.92;1.29] | **1.17** | [1.01;1.37] |  |  |  |  |  |  |
| **Cardiovascular mortality** | | | | | | | | | | | |
| Franek^14^ | 4,592 | 1.05 | [0.93;1.17] | 1.11 | [1.00;1.24] | **1.19** | [1.04;1.36] | **-** | - | - | - |
| Rådholm^16^ | 11,125 | 1.07 | [1.00;1.15] | **1.15** | [1.09;1.21] | **1.12** | [1.04;1.21] | **1.19** | [1.11;1.27] |  |  |
| **Hearth failure** | | | | | | | | | | | |
| Franek^14^ | 4,592 | **1.33** | [1.17;1.50] | **1.34** | [1.16;1.54] | **1.13** | [1.00;1.28] | **-** | - | - | - |
| German^7^ | 10,251 | **1.24** | [1.14;1.36] | **1.41** | [1.28;1.54] | - | - | - | - | - | - |
| **All -cause mortality** | | | | | | | | | | | |
| Franek^14^ | 4,592 | 1.05 | [0.96;1.15] | **1.10** | [1.00;1.20] | **1.08** | [1.00;1.18] | **-** | - | - | - |
| German^7^ | 10,251 | 1.04 | [0.97;1.12] | **1.18** | [1.10;1.27] | - | - | - | - | - | - |

*Data available only for the placebo group; **Abdominal TC; Bold character: p<0.05

**Table 3** – Main features of clinical trials included. Panel A (accuracy); panel B (risk of complications).

**A**

|  |  | **Mean parameters at entry** | | | | | |  |  |
| --- | --- | --- | --- | --- | --- | --- | --- | --- | --- |
| **First name** | **Follow-up**  (anni) | **Age**  (years) | **BMI**  (kg/m^2^) | **Waist circumference** (cm) | **Waist to hip ratio** | **Waist to height ratio** | **Study features** | | **Primary endpoint** |
| Bray^17^ | 3,2 | 53 | 32,8 | M:108 / F:102 | M:0,99 / F:0,88 | M:0,62 / F:0,62 | Case study derived from the Diabetes Prevention Program trial; subjects with BMI≥25 kg/m^2^ and IFG and/or IGT | | Incidence of diabetes |
| Franek^14^ | 5,4 | 66 | 32,3 | M:111 / F:107 | M:1,02 / F:0,95 | NR | Case study derived from REWIND trial; subjects with BMI≥25 kg/m^2^ and diabetes | | MACE, all-cause mortality; hearth failure |
| German^7^ | 3,7 | 63 | 32,2 | NR | NR | NR | Case study derived from ACCORD trial; subjects with BMI≥25 kg/m2 and diabetes | | MACE, all-cause mortality; hearth failure |
| Rådholm^16^ | 9,0 | 66 | 28,2 | M:100 / F:95 | M:1,0 / F:0,9 | M:0,59 / F:0,58 | Case study derived from ADVANCE ed ADVANCE-ON trials; subjects with BMI≥25 kg/m2 and diabetes | | MACE |
| Kadakia^15^ | 1,0 | 64 | 28,3 | All: 101 | NR | NR | Case study derived from MERLIN-TIMI 36 trial; subjects with BMI≥25 kg/m2 and previous CV events. | | MACE |

**B**

|  | **Mean parameters at entry** | | | | | |  |  |
| --- | --- | --- | --- | --- | --- | --- | --- | --- |
| **First name** | **Age**  (media/range; years) | **BMI**  (kg/m^2^) | **Waist circumference** (cm) | **Waist to hip ratio** | **Waist to height ratio** | **Study features** | | **Primary endpoint** |
| *Hayman^8^*^§^  *(1,394)* | NR / 20-79 | NR | NR | NR | NR | Working age people | | BMI accuracy |
| *Alammar^9^*^§^  *(942)* | 56 / 18-94 | 27,1 | NR | NR | NR | Adults subjects enrolled in Saudi Arabia (Primary care) | | BMI accuracy |
| *Alqarn^10^*^§^  *(319)** | < 60 (NR) | NR | NR | NR | NR | Adults subjects enrolled in Saudi Arabia (Primary care) | | BMI accuracy |
| *Batsis^13^*^§^  *(4,984)*** | ≥60 (NR) | 28,2 | NR | NR | NR | NHANES Study 1999-2004; subjects ≥60 years of age | | BMI accuracy |
| Porto^12§^  *(3,822)* | 37 (24-49) | 26,5 | NR | NR | NR | Military men | | BMI accuracy |
| Vasconcelos^5§^  *(180)* | 68 (NR) | 26,9 | NR | NR | NR | Healthy elderly people. | | BMI accuracy |
| Zwierzchowska^11§^  *(87)* | 62 (40-80) | 28,5 | 94 | 0,88 | 0,59 | Women ≥40 years of age | | Anthropometric indexes accuracy |

MACE: Major CArdiovascular Events

**Table 4** –GRADE assessment of the quality of retrieved evidence.

| **Certainty assessment** | | | | | | | **Summary of findings** | | | | |
| --- | --- | --- | --- | --- | --- | --- | --- | --- | --- | --- | --- |
| **Participants (studies) Follow up** | **Risk of bias** | **Inconsistency** | **Indirectness** | **Imprecision** | **Publication bias** | **Overall certainty of evidence** | **Study event rates (%)** | | **Relative effect (95% CI)** | **Anticipated absolute effect** | |
|  |  |  |  |  |  |  | **With PBO** | **With BMI 30-35** |  | **Risk with PBO** | **Risk difference with BMI 30-35** |
| **Diagnostic accuracy** | | | | | | | | | | | |
| 32,859 (5 observ.) | serious | not serious | not serious | not serious | none | ⨁⨁-- Low^a^ | - | - | - | - | - |
| **Risk of diabetes** | | | | | | | | | | | |
| 331 (1 observ.) | serious^a^ | not serious | not serious | very serious | none | ⨁--- Very low ^a,b^ | - | - | - | - | - |
| **Risk of cardiovascular events** | | | | | | | | | | | |
| 32,528 (4 observ.) | serious^a^ | not serious | not serious | not serious | none | ⨁⨁-- Low^a^ | - | - | - | - | - |
| **Cardiovascular mortality** | | | | | | | | | | | |
| 15,717 (2 observ.) | serious^a^ | not serious | not serious | not serious | none | ⨁⨁-- Low^a^ | - | - | - | - | - |
| **Hearth failure** | | | | | | | | | | | |
| 14,843 (2 observ.) | serious^a^ | not serious | not serious | not serious | none | ⨁⨁-- Low^a^ | - | - | - | - | - |
| **All – cause mortality** | | | | | | | | | | | |
| 14,843 (2 observ.) | serious^a^ | not serious | not serious | not serious | none | ⨁⨁-- Low^a^ | - | - | - | - | - |

a. observational studies: b. data derived from one single study.

**Figures**

Figure 1 – Metanalysis of odds ratio for incident diabetes.

**BMI**

**Waist circumference**

**Waist-****to-Height ratio**

**Fat mass**

**Waist-to- hip ratio**

Figure 2 – Metanalysis of odds ratio for incident MACE.

**BMI**

**Waist circumference**

**Waist-to-Hip ratio**

Figure 3 – Metanalysis of odds ratio for cardiovascular death risk.

**BMI**

**

Waist circumference **

Waist-to-Hip ratio

Figure 4 – Metanalysis of odds ratio for hospitalisation for hearth failure.

**BMI**

**

Waist circumference

Figure 5 – Metanalysis of odds ratio for all-cause mortality risk.

**BMI**

**

Waist circumference

**PICO 2**

*Pubmed: # 4072*

*Search: obesity and (BMI or waist or "body fat" or "body composition" or "lean mass") and (DEXA or TC) Filters: Randomized Controlled Trial*

*(("obeses"[All Fields] OR "obesity"[MeSH Terms] OR "obesity"[All Fields] OR "obese"[All Fields] OR "obesities"[All Fields] OR "obesity s"[All Fields]) AND ("BMI"[All Fields] OR ("waist"[All Fields] OR "waists"[All Fields]) OR "body fat"[All Fields] OR "body composition"[All Fields] OR "lean mass"[All Fields]) AND ("diabete"[All Fields] OR "diabetes mellitus"[MeSH Terms] OR ("diabetes"[All Fields] AND "mellitus"[All Fields]) OR "diabetes mellitus"[All Fields] OR "diabetes"[All Fields] OR "diabetes insipidus"[MeSH Terms] OR ("diabetes"[All Fields] AND "insipidus"[All Fields]) OR "diabetes insipidus"[All Fields] OR "diabetic"[All Fields] OR "diabetics"[All Fields] OR "diabets"[All Fields] OR ("cardiovascular system"[MeSH Terms] OR ("cardiovascular"[All Fields] AND "system"[All Fields]) OR "cardiovascular system"[All Fields] OR "cardiovascular"[All Fields] OR "cardiovasculars"[All Fields]) OR ("cancer s"[All Fields] OR "cancerated"[All Fields] OR "canceration"[All Fields] OR "cancerization"[All Fields] OR "cancerized"[All Fields] OR "cancerous"[All Fields] OR "neoplasms"[MeSH Terms] OR "neoplasms"[All Fields] OR "cancer"[All Fields] OR "cancers"[All Fields]) OR ("mortality"[MeSH Terms] OR "mortality"[All Fields] OR "mortalities"[All Fields] OR "mortality"[MeSH Subheading]) OR "sleep apnea"[All Fields] OR "steato-hepatitis"[All Fields])) AND (randomizedcontrolledtrial[Filter])*

*Translations*

*obesity: "obeses"[All Fields] OR "obesity"[MeSH Terms] OR "obesity"[All Fields] OR "obese"[All Fields] OR "obesities"[All Fields] OR "obesity's"[All Fields]*

*waist: "waist"[All Fields] OR "waists"[All Fields]*

*diabetes: "diabete"[All Fields] OR "diabetes mellitus"[MeSH Terms] OR ("diabetes"[All Fields] AND "mellitus"[All Fields]) OR "diabetes mellitus"[All Fields] OR "diabetes"[All Fields] OR "diabetes insipidus"[MeSH Terms] OR ("diabetes"[All Fields] AND "insipidus"[All Fields]) OR "diabetes insipidus"[All Fields] OR "diabetic"[All Fields] OR "diabetics"[All Fields] OR "diabets"[All Fields]*

*cardiovascular: "cardiovascular system"[MeSH Terms] OR ("cardiovascular"[All Fields] AND "system"[All Fields]) OR "cardiovascular system"[All Fields] OR "cardiovascular"[All Fields] OR "cardiovasculars"[All Fields]*

*cancer: "cancer's"[All Fields] OR "cancerated"[All Fields] OR "canceration"[All Fields] OR "cancerization"[All Fields] OR "cancerized"[All Fields] OR "cancerous"[All Fields] OR "neoplasms"[MeSH Terms] OR "neoplasms"[All Fields] OR "cancer"[All Fields] OR "cancers"[All Fields]*

*mortality: "mortality"[MeSH Terms] OR "mortality"[All Fields] OR "mortalities"[All Fields] OR "mortality"[Subheading]*

Tables

Table 5 – Predictive value of single anthropometric indexes for different obesity – related comorbidities. Relative risk for every incremental point of single parameters. Analysis performed on RCT or sub-analysis of clinical trials.

| **First author** | **n. pts** | **Body Fat** (%; visceral) | |
| --- | --- | --- | --- |
|  |  | OR | *95%, CI* |
|  |  | ***Risk of incident diabetes*** | |
| Bray^17^  Men  Women | Placebo  108 | **1.48**  1.28 | [1.02;2.14]  [0.96;1.70] |
| Bray^17^  Men  Women | Metformin  112 | 1.67  1.16 | [0.95;2.93]  [0.82;1.64] |
| Bray^17^  Men  Women | Lifestyle  111 | 1.61  1.40 | [0.82;3.15]  [0.92;2.14] |

**Table 6** – Main features of clinical trials included.

|  |  | **Mean parameters at entry** | | | | | | OR/AUC | *95%, CI* |
| --- | --- | --- | --- | --- | --- | --- | --- | --- | --- |
| **First author** | **Follow-up**  (years) | **Age**  (years) | **BMI**  (kg/m^2^) | **Waist circumference** (cm) | **Waist-to-Hip ratio** | **Waist-to-Height ratio** | **Study features** | | **Primary endpoint** |
| Bray^17^ | 3,2 | 53 | 32,8 | M:108 / F:102 | M:0,99 / F:0,88 | M:0,62 / F:0,62 | Case study derived from the Diabetes Prevention Program trial; subjects with BMI≥25 kg/m2 and IGT. | | Incident diabetes |

**Table 7** – GRADE assessment of the quality of retrieved evidence.

| **Certainty assessment** | | | | | | | **Summary of** **findings** | | | | |
| --- | --- | --- | --- | --- | --- | --- | --- | --- | --- | --- | --- |
| **Participants**  **(studies)**  **Follow up (CI)** | **Risk of bias** | **Inconsistency** | **Indirectness** | **Imprecision** | **Publication bias** | **Overall certainty of evidence** | **Study event rates (%)** | | **Relative effect**  **(95% CI)** | **Anticipated absolute effect** | |
|  |  |  |  |  |  |  | **With PBO** | **With BMI 30-35** |  | **Risk with PBO** | **Risk difference with BMI 30-35** |
| **Diagnostic accuracy** | | | | | | | | | | | |
| 331 (1 obser.) | serious^a^ | not serious | not serious | very serious^b^ | none | ⨁--- very low^a,b^ | - | - | - | - | - |

Pharmacoeconomic evidence - PICO 1-2

**Search string** *(until 21/05/2025)*

*Pubmed: # 4072*

*Search: obesity and (BMI or waist or "body fat" or "body composition" or "lean mass") and (diabetes or cardiovascular or cancer or mortality or "sleep apnea" or steato-hepatitis) and economic. Filters: Randomized Controlled Trial*

*(("obeses"[All Fields] OR "obesity"[MeSH Terms] OR "obesity"[All Fields] OR "obese"[All Fields] OR "obesities"[All Fields] OR "obesity s"[All Fields]) AND ("BMI"[All Fields] OR ("waist"[All Fields] OR "waists"[All Fields]) OR "body fat"[All Fields] OR "body composition"[All Fields] OR "lean mass"[All Fields]) AND ("diabete"[All Fields] OR "diabetes mellitus"[MeSH Terms] OR ("diabetes"[All Fields] AND "mellitus"[All Fields]) OR "diabetes mellitus"[All Fields] OR "diabetes"[All Fields] OR "diabetes insipidus"[MeSH Terms] OR ("diabetes"[All Fields] AND "insipidus"[All Fields]) OR "diabetes insipidus"[All Fields] OR "diabetic"[All Fields] OR "diabetics"[All Fields] OR "diabets"[All Fields] OR ("cardiovascular system"[MeSH Terms] OR ("cardiovascular"[All Fields] AND "system"[All Fields]) OR "cardiovascular system"[All Fields] OR "cardiovascular"[All Fields] OR "cardiovasculars"[All Fields]) OR ("cancer s"[All Fields] OR "cancerated"[All Fields] OR "canceration"[All Fields] OR "cancerization"[All Fields] OR "cancerized"[All Fields] OR "cancerous"[All Fields] OR "neoplasms"[MeSH Terms] OR "neoplasms"[All Fields] OR "cancer"[All Fields] OR "cancers"[All Fields]) OR ("mortality"[MeSH Terms] OR "mortality"[All Fields] OR "mortalities"[All Fields] OR "mortality"[MeSH Subheading]) OR "sleep apnea"[All Fields] OR "steato-hepatitis"[All Fields])) AND (randomizedcontrolledtrial[Filter])*

*Translations*

*obesity: "obeses"[All Fields] OR "obesity"[MeSH Terms] OR "obesity"[All Fields] OR "obese"[All Fields] OR "obesities"[All Fields] OR "obesity's"[All Fields]*

*waist: "waist"[All Fields] OR "waists"[All Fields]*

*diabetes: "diabete"[All Fields] OR "diabetes mellitus"[MeSH Terms] OR ("diabetes"[All Fields] AND "mellitus"[All Fields]) OR "diabetes mellitus"[All Fields] OR "diabetes"[All Fields] OR "diabetes insipidus"[MeSH Terms] OR ("diabetes"[All Fields] AND "insipidus"[All Fields]) OR "diabetes insipidus"[All Fields] OR "diabetic"[All Fields] OR "diabetics"[All Fields] OR "diabets"[All Fields]*

*cardiovascular: "cardiovascular system"[MeSH Terms] OR ("cardiovascular"[All Fields] AND "system"[All Fields]) OR "cardiovascular system"[All Fields] OR "cardiovascular"[All Fields] OR "cardiovasculars"[All Fields]*

*cancer: "cancer's"[All Fields] OR "cancerated"[All Fields] OR "canceration"[All Fields] OR "cancerization"[All Fields] OR "cancerized"[All Fields] OR "cancerous"[All Fields] OR "neoplasms"[MeSH Terms] OR "neoplasms"[All Fields] OR "cancer"[All Fields] OR "cancers"[All Fields]*

*mortality: "mortality"[MeSH Terms] OR "mortality"[All Fields] OR "mortalities"[All Fields] OR "mortality"[Subheading]*

No studies found.

**PICO 3-6**

**Search string** *(until 19/03/2025): (Overweight or Obesit**y) AND (ketogenic or very-low-calorie or Mediterranean or balanced diet)*

*Pubmed: #1,061:*

*(("overweight"[MeSH Terms] OR "overweight"[All Fields] OR "overweighted"[All Fields] OR "overweightness"[All Fields] OR "overweights"[All Fields] OR ("obeses"[All Fields] OR "obesity"[MeSH Terms] OR "obesity"[All Fields] OR "obese"[All Fields] OR "obesities"[All Fields] OR "obesity s"[All Fields])) AND ("ketogenic"[All Fields] OR ("very-low"[All Fields] AND ("calorie"[All Fields] OR "calories"[All Fields] OR "calory"[All Fields])) OR ("mediterranean"[All Fields] OR "mediterraneans"[All Fields]) OR (("balance"[All Fields] OR "balanced"[All Fields] OR "balances"[All Fields] OR "balancing"[All Fields]) AND ("diet"[MeSH Terms] OR "diet"[All Fields])))) AND (randomizedcontrolledtrial[Filter])*

*Translations*

*Overweight: "overweight"[MeSH Terms] OR "overweight"[All Fields] OR "overweighted"[All Fields] OR "overweightness"[All Fields] OR "overweights"[All Fields]*

*Obesity: "obeses"[All Fields] OR "obesity"[MeSH Terms] OR "obesity"[All Fields] OR "obese"[All Fields] OR "obesities"[All Fields] OR "obesity's"[All Fields]*

*calorie: "calorie"[All Fields] OR "calories"[All Fields] OR "calory"[All Fields]*

*Mediterranean: "mediterranean"[All Fields] OR "mediterranean's"[All Fields] OR "mediterraneans"[All Fields]*

*balanced: "balance"[All Fields] OR "balanced"[All Fields] OR "balances"[All Fields] OR "balancing"[All Fields]*

*diet: "diet"[MeSH Terms] OR "diet"[All Fields]*

*Embase: #436:*

*('overweight'/exp OR overweight OR 'obesity'/exp OR obesity) AND (ketogenic OR 'very-low calorie' OR ('very low' AND ('calorie'/exp OR calorie)) OR 'mediterranean'/exp OR mediterranean OR 'balanced diet'/exp OR 'balanced diet' OR (balanced AND ('diet'/exp OR diet))) AND [embase]/lim NOT ([embase]/lim AND [medline]/lim) AND 'randomized controlled trial'/de*

No studies were retrieved.

Figures

**PICO 3**

**Figure 6** – Comparison between structured education programs and unstructured education programs on BMI (WMD) at study end.

**
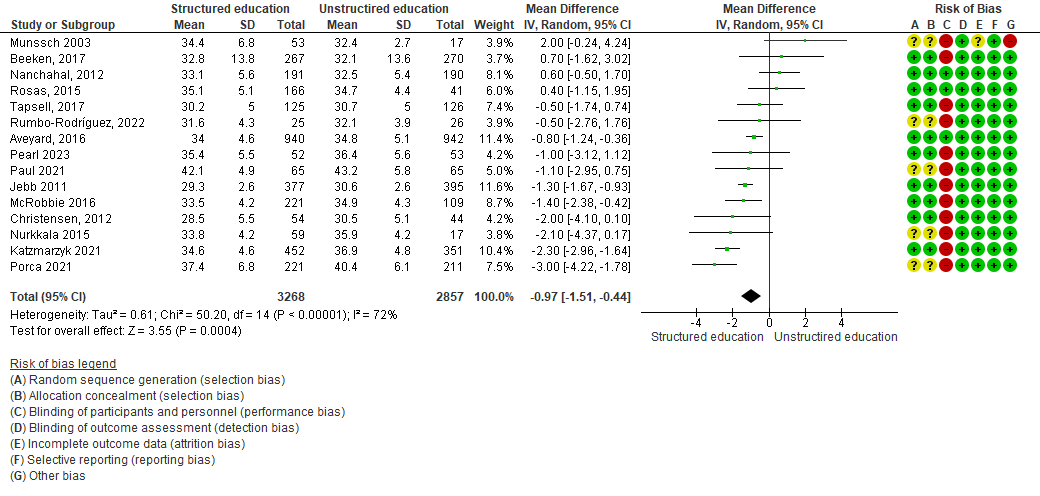
**

**Figure 7** – Comparison between structured education programs and unstructured education programs on waist circumference (WC) at study end.

**
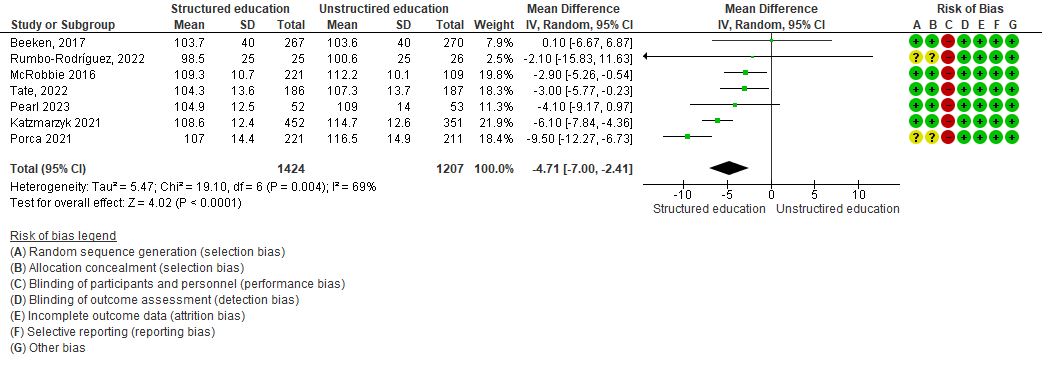
**

**Figure 8 –** Comparison between structured education programs and unstructured education programs on percentage weight loss (TBWL%; WMD) at study end.

**
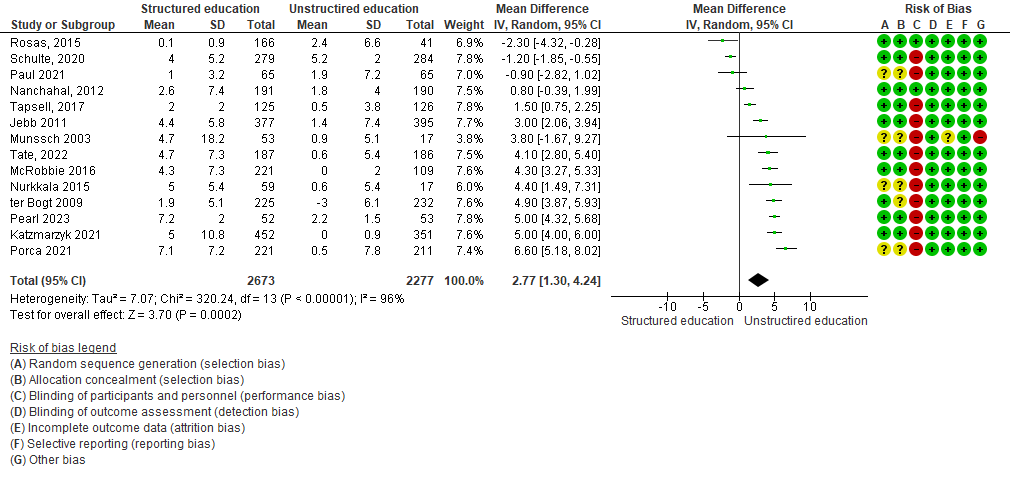
**

**Figure 9** – Comparison between structured education programs and unstructured education programs on probability (OR) to reach 5% (Panel A) and 10% (Panel B) weight loss from baseline at study end.

**A**

**
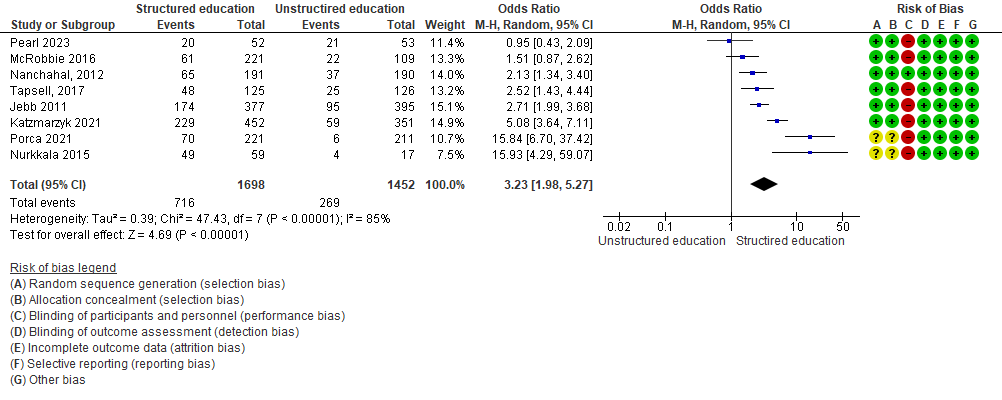
**

**B**

**
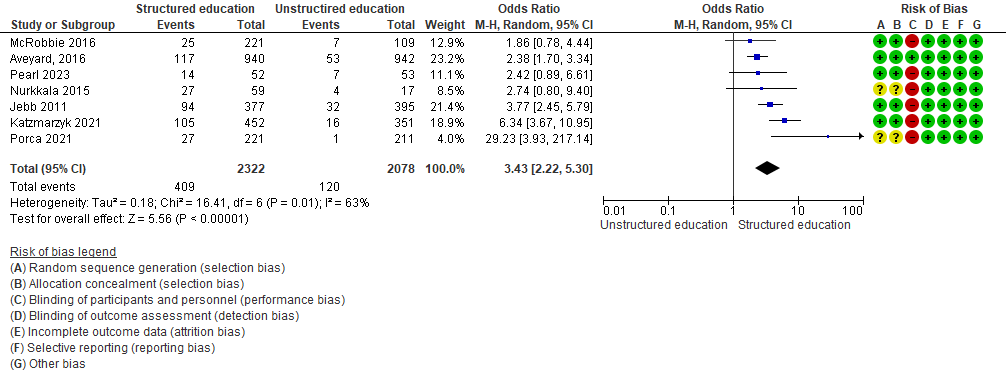
**

**Figure 10 -** Comparison between structured education programs and unstructured education programs on percentage fat mass loss (WMD) at study end.

**
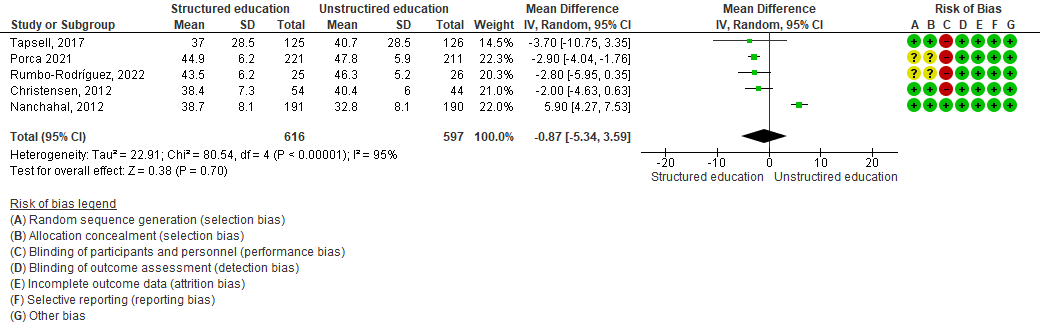
**

**PICO 4**

**Figure 11** – Comparison between cognitive behavioural therapy and other kinds of educational programs on BMI (WMD) at study end.


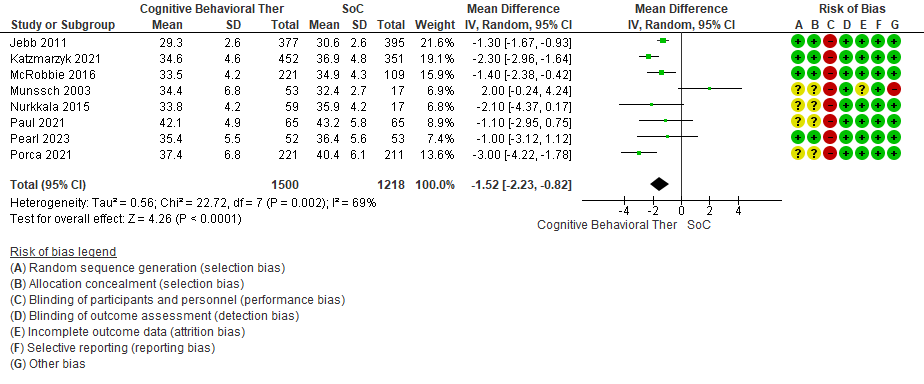


**Figure 12** – Comparison between cognitive behavioural therapy and other kinds of educational programs on waist circumference (WMD) at study end.


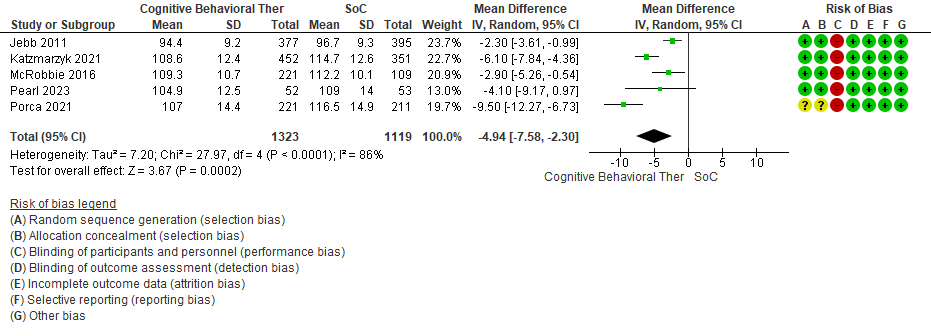


**Figure 13** – Comparison between cognitive behavioral therapy and other kinds of educational programs on percentage weight loss (WMD) at study end.

**
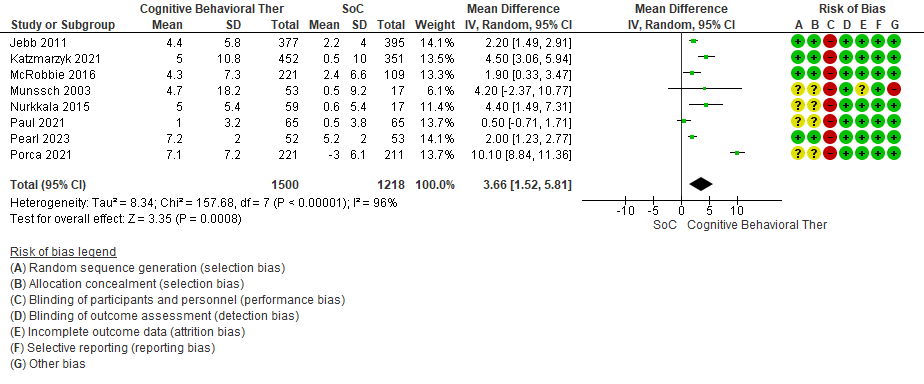
**

**Figure 14** – Comparison between cognitive behavioural therapy and other kinds of educational programs on probability (OR) to reach 5% (Panel A) and 10% (Panel B) weight loss from baseline at study end.

**A**

**
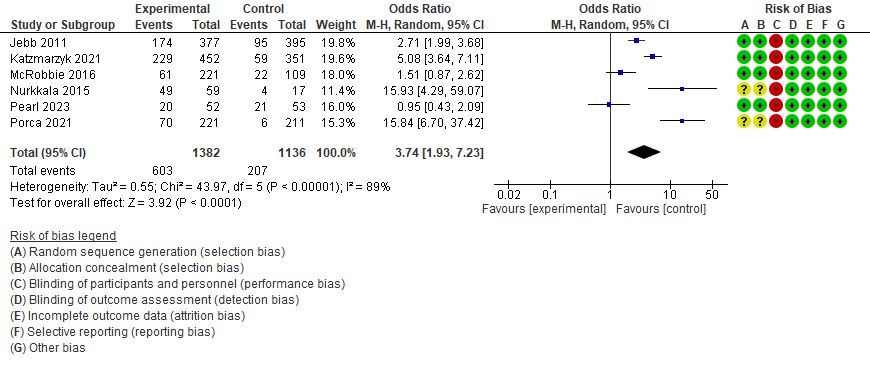
**

**B**

**
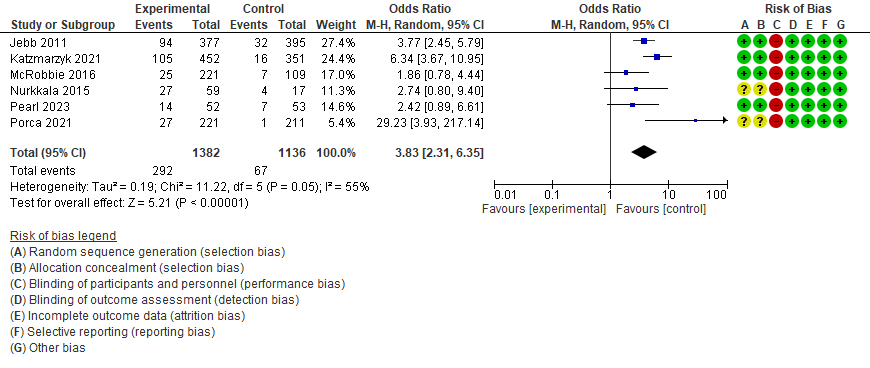
**

**PICO 5**

**Figure 15** – Comparison between ketogenic and Mediterranean diets on fat mass percentage at study end.


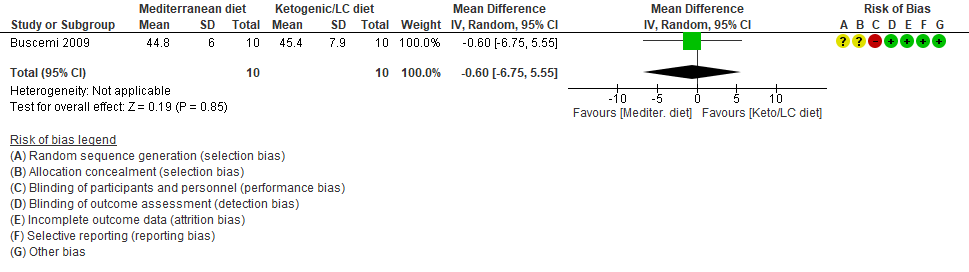


**Figure 16** – Comparison between ketogenic and Mediterranean diets on BMI at study end.


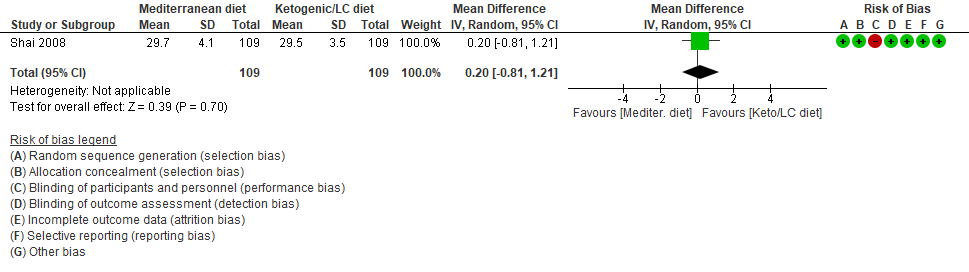


**Figure 17** – Comparison between ketogenic and Mediterranean diets on waist circumference at study end.


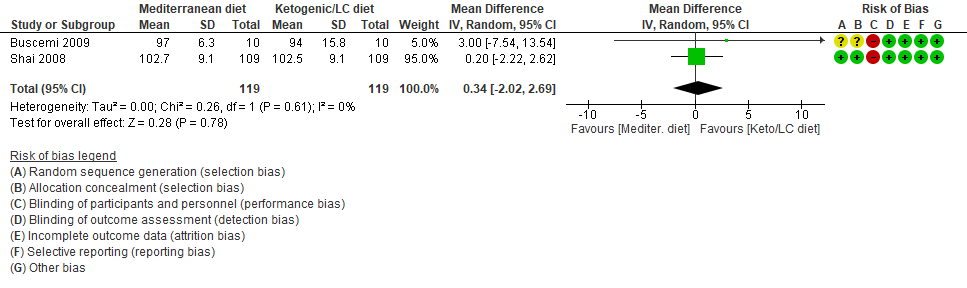


**Figure 18** – Comparison between ketogenic and Mediterranean diets on percentage weight loss at study end.


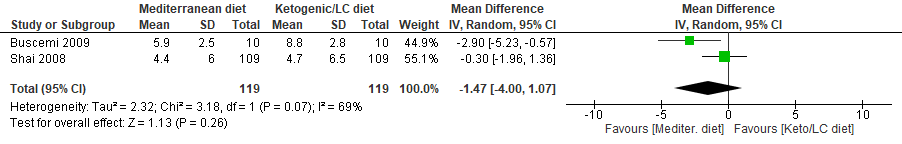


**PICO 6**

**Figure 19** – Comparison between educational programs including combined physical exercises (aerobic and resistance) and educational programs including aerobic exercises, on BMI (WMD) at study end.


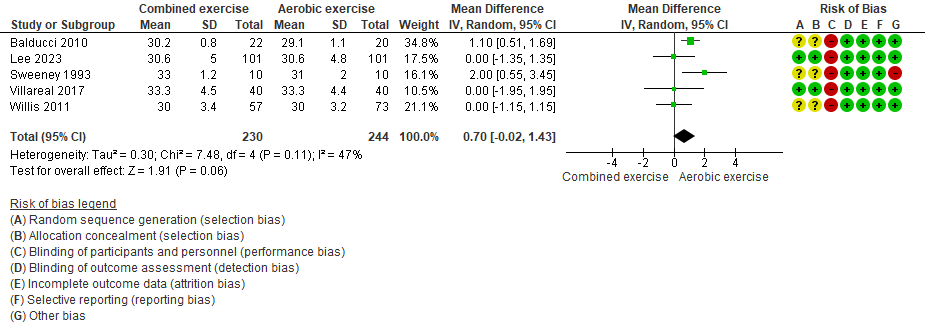


**Figure 20** – Comparison between educational programs including combined physical exercises (aerobic and resistance) and educational programs including aerobic exercises, on waist circumference (WMD) at study end.


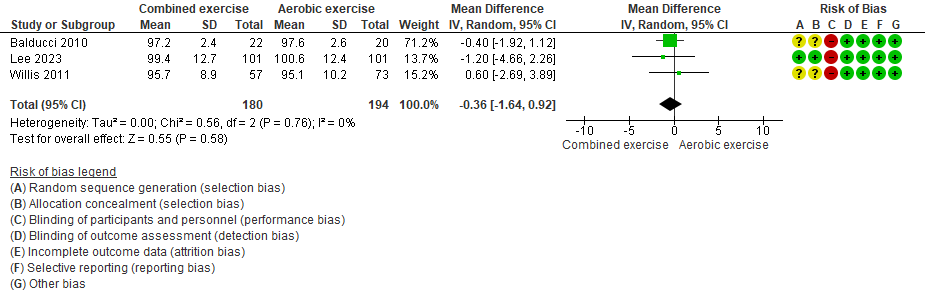


**Figure 21** – Comparison between educational programs including combined physical exercises (aerobic and resistance) and educational programs including aerobic exercises, on fat mass percentage at study end.


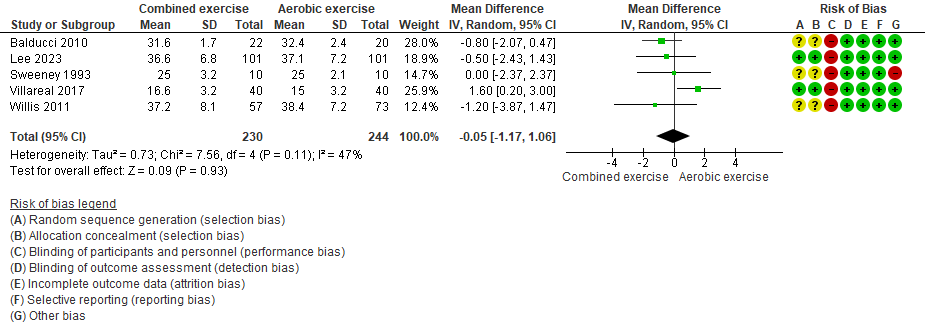


Tables

*Table 8-* *PICO 3: main features of included studies.*

| **N** | **Study Name** | **Intervention** | **Comparator** | **N. pat.**  **Interv.** | **N. pat.**  **Comp.** | **Trial duration**  (weeks) | **BMI min.**  (Kg/m^2^) | **BMI max.**  (Kg/m^2^) | **Age max.**  (years) | **Mean BMI**  (Kg/m^2^) | **Mean Age**  (years) |
| --- | --- | --- | --- | --- | --- | --- | --- | --- | --- | --- | --- |
| 1 | Aveyard^31^ | Structured education | Unstructured education | 940 | 942 | 52 | 25 | NR | NR | 34,9 | 56 |
| 2 | Beeken^32^ | Structured education | Unstructured education | 267 | 270 | 104 | 30 | NR | NR | 35,0 | 60 |
| 3 | Christensen^33^ | Structured education | Unstructured education | 54 | 44 | 52 | 25 | NR | NR | 30,5 | NR |
| 4 | Jebb^23^ | Cognitive behavioral therapy | Unstructured education | 377 | 395 | 52 | 27 | 35 | NR | 31,4 | 47 |
| 5 | Katzmarzyk^24^ | Cognitive behavioral therapy | Unstructured education | 452 | 351 | 104 | 30 | 50 | 75 | 37,0 | 49 |
| 6 | McRobbie^25^ | Cognitive behavioral therapy | Unstructured education | 221 | 109 | 52 | 28 | 45 | NR | 35,3 | 46 |
| 7 | Munssch^26^ | Cognitive behavioral therapy | Unstructured education | 53 | 17 | 52 | 30 | NR | NR | 36,0 | 45 |
| 8 | Nanchahal^34^ | Structured education | Unstructured education | 191 | 190 | 52 | 25 | NR | NR | 33,5 | 49 |
| 9 | Nurkkala^27^ | Cognitive behavioral therapy | Unstructured education | 59 | 17 | 156 | 30 | NR | 65 | 34,3 | 46 |
| 10 | Paul^28^ | Cognitive behavioral therapy | Unstructured education | 65 | 65 | 52 | 30 | NR | 65 | 43,0 | 42 |
| 11 | Pearl^29^ | Cognitive behavioral therapy | Unstructured education | 52 | 53 | 72 | 27 | NR | NR | 38,0 | 49 |
| 12 | Porca^30^ | Cognitive behavioral therapy | Unstructured education | 221 | 211 | 52 | 30 | NR | 77 | 39,9 | 49 |
| 13 | Rosas^35^ | Structured education | Unstructured education | 166 | 41 | 104 | 30 | 60 | NR | 35,6 | 47 |
| 14 | Rumbo-Rodríguez^36^ | Structured education | Unstructured education | 25 | 26 | 104 | 25 | NR | NR | 32,4 | 65 |
| 15 | Schulte^37^ | Structured education | Unstructured education | 279 | 284 | 52 | 27 | 35 | NR | 37,1 | 55 |
| 16 | Tapsell^38^ | Structured education | Unstructured education | 125 | 126 | 52 | 25 | 40 | 54 | 32,0 | 45 |
| 17 | Tate^39^ | Structured education | Unstructured education | 187 | 186 | 52 | 25 | 45 | 75 | 33,8 | NR |
| 18 | ter Bogt^40^ | Structured education | Unstructured education | 225 | 232 | 156 | 25 | NR | 70 | 29,5 | 56 |

*Table 9– PICO 4:* *main features of included studies.*

| **N** | **Study Name** | **Intervention** | **Comparator** | **N. pat.**  **Interv.** | **N. pat.**  **Comp.** | **Trial duration**  (weeks) | **BMI min.**  (Kg/m^2^) | **BMI max.**  (Kg/m^2^) | **Age max.**  (years) | **Mean BMI**  (Kg/m^2^) | **Mean Age**  (years) |
| --- | --- | --- | --- | --- | --- | --- | --- | --- | --- | --- | --- |
| 1 | *Jebb^23^* | CBT | SoC | 377 | 395 | 52 | 27 | 35 | NR | 31,4 | 47,0 |
| 2 | *Katzmarzyk^24^* | CBT | SoC | 452 | 351 | 26 | 30 | 50 | 75 | 37,0 | 49,0 |
| 3 | *McRobbie^25^* | CBT | SoC | 221 | 109 | 8 | 28 | 45 | NR | 35,3 | 46,0 |
| 4 | *Munssch^26^* | CBT | SoC | 53 | 17 | 16 | 30 | NR | NR | 36,0 | 45,2 |
| 5 | *Nurkkala^27^* | CBT | SoC | 59 | 17 | 48 | 30 | NR | 65 | 34,3 | 46,0 |
| 6 | *Paul^28^* | CBT | SoC | 65 | 65 | 10 | 30 | NR | 65 | 43,0 | 41,7 |
| 7 | *Pearl^29^* | CBT | SoC | 52 | 53 | 20 | 27 | NR | NR | 38,0 | 49,0 |
| 8 | *Porca^30^* | CBT | SoC | 221 | 211 | 6 | 30 | NR | 77 | 39,9 | 48,8 |

*CBT: cognitive behavioral therapy; SoC: Standard of Care.*

*Table 10 – PICO 5:* *main features of included studies.*

| **N** | **Study Name** | **Intervention** | **Comparator** | **N. pat.**  **Interv.** | **N. pat.**  **Comp.** | **Trial duration**  (weeks) | **BMI min.**  (Kg/m^2^) | **BMI max.**  (Kg/m^2^) | **Age max.**  (years) | **Mean BMI**  (Kg/m^2^) | **Mean Age**  (years) |
| --- | --- | --- | --- | --- | --- | --- | --- | --- | --- | --- | --- |
| 1 | *Buscemi^51^* | Ketogenic diet | Mediterranean diet | 10 | 10 | 8 | 27 | 35 | 50,00 | 34,2 | 38,00 |
| 2 | *Shai^52^* | Ketogenic diet | Mediterranean diet | 109 | 109 | 104 | 27 | NR | 65,00 | 31,0 | 52,00 |

*Table 11– PICO 6: main features of included studies.*

| **N** | **Study Name** | **Intervention** | **Comparator** | **N. pat.**  **Interv.** | **N. pat.**  **Comp.** | **Trial duration**  (weeks) | **BMI min.**  (Kg/m^2^) | **BMI max.**  (Kg/m^2^) | **Age max.**  (years) | **Mean BMI**  (Kg/m^2^) | **Mean Age**  (years) |
| --- | --- | --- | --- | --- | --- | --- | --- | --- | --- | --- | --- |
| 1 | *Balducci* | Combined physical exercise | Aerobic exercise | 22 | 20 | 52 | 27 | 40 | 75 | 30,0 | 62,50 |
| 2 | *Lee* | Combined physical exercise | Aerobic exercise | 101 | 101 | 52 | 25 | 40 | 70 | 31,1 | 50,50 |
| 3 | *Ramos-Campo* | Combined physical exercise | Aerobic exercise | 30 | 30 | 24 | 30 | 35 | 50 | NR | 38,00 |
| 4 | *Sweeney* | Combined physical exercise | Aerobic exercise | 10 | 10 | 26 | NR | NR | NR | 36,0 | 36,00 |
| 5 | *Villareal* | Combined physical exercise | Aerobic exercise | 40 | 40 | 26 | 30 | NR | NR | 35,8 | 70,00 |
| 6 | *Willis* | Combined physical exercise | Aerobic exercise | 57 | 73 | 36 | 25 | 35 | 70 | NR | NR |

**Table 12** – GRADE assessment of the quality of retrieved evidence.

| **Certainty assessment** | | | | | | | **Summary of findings** | | | | |
| --- | --- | --- | --- | --- | --- | --- | --- | --- | --- | --- | --- |
| **Participants (studies) follow up** | **Risk of bias** | **Inconsistency** | **Indirectness** | **Imprecision** | **Publication bias** | **Overall certainty of evidence** | **Study event rates (%)** | | **Relative effect (95% CI)** | **Anticipated absolute effect** | |
|  |  |  |  |  |  |  | **With comparison** | **With intervention** |  | **Risk with comparison** | **Risk difference between comparison and intervention** |
| **PICO 3 - BMI** | | | | | | | | | | | |
| 6125 (15 RCT) | serious^a^ | serious^b^ | not serious | not serious | strong association | ⨁⨁⨁- Moderate^a,b^ | 2857 | 3268 | - | - | MD 0.97 fewer (from 1.51 fewer to 0.44 fewer) |
| **PICO 4 - BMI** | | | | | | | | | | | |
| 2718 (8 RCT) | serious^a^ | serious^b^ | not serious | not serious | strong association | ⨁⨁⨁- Moderate^a,b^ | 1218 | 1500 | - | - | MD 1.52 fewer (from 2.23 fewer to 0.82 fewer) |
| **PICO 5 - BMI** | | | | | | | | | | | |
| 218 (2 RCT) | serious^a^ | not serious | not serious | very serious^c^ | none | ⨁⨁⨁- Very low^a,c^ | 119 | 119 | - | - | MD 0.20 fewer (from 0.81 fewer to 1.21 higher) |
| **PICO 6 – BMI** | | | | | | | | | | | |
| 474 (5 RCTs) | serious^a^ | not serious | not serious | serious^c^ | none | ⨁⨁◯◯ Low^a,c^ | 244 | 230 | - | - | MD 0.7 higher (from 0.02 fewer to 1.43 higher) |

a) Studies with bias (open label); b) High heterogeneity I^2^>50%; c) Studies including few subjects.

***Pharmacoeconomic evidence***

***Search s******tring*** *(until 19/03/2025): (economic or cost or cost-saving or cost-effectiveness) AND (Overweight or Obesity) AND (ketogenic or very-low-calorie or Mediterranean or balanced diet)*

*Pubmed: #612:*

*Search: (economic or cost or cost-saving or cost-effectiveness) AND (Overweight or Obesity) AND (ketogenic or very-low-calorie or Mediterranean or balanced diet)*

*("economical"[All Fields] OR "economics"[MeSH Terms] OR "economics"[All Fields] OR "economic"[All Fields] OR "economically"[All Fields] OR "economics"[MeSH Subheading] OR "economization"[All Fields] OR "economize"[All Fields] OR "economized"[All Fields] OR "economizes"[All Fields] OR "economizing"[All Fields] OR ("economics"[MeSH Subheading] OR "economics"[All Fields] OR "cost"[All Fields] OR "costs and cost analysis"[MeSH Terms] OR ("costs"[All Fields] AND "cost"[All Fields] AND "analysis"[All Fields]) OR "costs and cost analysis"[All Fields]) OR ("cost savings"[MeSH Terms] OR ("cost"[All Fields] AND "savings"[All Fields]) OR "cost savings"[All Fields] OR ("cost"[All Fields] AND "saving"[All Fields]) OR "cost saving"[All Fields]) OR ("cost effectiveness analysis"[MeSH Terms] OR ("cost effectiveness"[All Fields] AND "analysis"[All Fields]) OR "cost effectiveness analysis"[All Fields] OR ("cost"[All Fields] AND "effectiveness"[All Fields]) OR "cost effectiveness"[All Fields])) AND ("overweight"[MeSH Terms] OR "overweight"[All Fields] OR "overweighted"[All Fields] OR "overweightness"[All Fields] OR "overweights"[All Fields] OR ("obeses"[All Fields] OR "obesity"[MeSH Terms] OR "obesity"[All Fields] OR "obese"[All Fields] OR "obesities"[All Fields] OR "obesity s"[All Fields])) AND ("ketogenic"[All Fields] OR "very-low-calorie"[All Fields] OR ("mediterranean"[All Fields] OR "mediterraneans"[All Fields]) OR (("balance"[All Fields] OR "balanced"[All Fields] OR "balances"[All Fields] OR "balancing"[All Fields]) AND ("diet"[MeSH Terms] OR "diet"[All Fields])))*

*Translations*

*economic: "economical"[All Fields] OR "economics"[MeSH Terms] OR "economics"[All Fields] OR "economic"[All Fields] OR "economically"[All Fields] OR "economics"[Subheading] OR "economization"[All Fields] OR "economize"[All Fields] OR "economized"[All Fields] OR "economizes"[All Fields] OR "economizing"[All Fields]*

*cost: "economics"[Subheading] OR "economics"[All Fields] OR "cost"[All Fields] OR "costs and cost analysis"[MeSH Terms] OR ("costs"[All Fields] AND "cost"[All Fields] AND "analysis"[All Fields]) OR "costs and cost analysis"[All Fields]*

*cost-saving: "cost savings"[MeSH Terms] OR ("cost"[All Fields] AND "savings"[All Fields]) OR "cost savings"[All Fields] OR ("cost"[All Fields] AND "saving"[All Fields]) OR "cost saving"[All Fields]*

*cost-effectiveness: "cost-effectiveness analysis"[MeSH Terms] OR ("cost-effectiveness"[All Fields] AND "analysis"[All Fields]) OR "cost-effectiveness analysis"[All Fields] OR ("cost"[All Fields] AND "effectiveness"[All Fields]) OR "cost effectiveness"[All Fields]*

*Overweight: "overweight"[MeSH Terms] OR "overweight"[All Fields] OR "overweighted"[All Fields] OR "overweightness"[All Fields] OR "overweights"[All Fields]*

*Obesity: "obeses"[All Fields] OR "obesity"[MeSH Terms] OR "obesity"[All Fields] OR "obese"[All Fields] OR "obesities"[All Fields] OR "obesity's"[All Fields]*

*Mediterranean: "mediterranean"[All Fields] OR "mediterranean's"[All Fields] OR "mediterraneans"[All Fields]*

*balanced: "balance"[All Fields] OR "balanced"[All Fields] OR "balances"[All Fields] OR "balancing"[All Fields]*

*diet: "diet"[MeSH Terms] OR "diet"[All Fields]*

GRADE evaluation of single critical outcome

Table 17

PICO 7

| **Certainty assessment** | | | | | | | | | | | **№ subjects** | | | **Effect** | | **Overall certainty of evidence** | |  |  |
| --- | --- | --- | --- | --- | --- | --- | --- | --- | --- | --- | --- | --- | --- | --- | --- | --- | --- | --- | --- |
| **Participants**  **(studies)**  **follow up** | **Study design** | **Risk of bias** | **Inconsistency** | | **Indirectness** | | | **Imprecision** | | **Other considerations** | **Intervention** | | **Comparator** | **Relative (95% CI)** | **Absolute (95% CI)** |  |  |  |  |
| **Weight loss- Liraglutide** | | | | | | | | | | | | | | | | | | |  |
| *62*  *(1 RCT)* | *RCT* | *Not serious* | *Not serious* | | *Not serious* | | | *Very serious^a^* | | *Strong association* | *23* | | *39* | *-* | *MD 13.9 higher (from 10.8 higher to 17.0 higher)* | *⨁⨁◯◯*  *Low* | |  |  |
| **Weight loss - Semaglutide** | | | | | | | | | | | | | | | | | | |  |
| *91*  *(1 RCT)* | | *RCT* | *Not serious* | | *Not serious* | | *Not serious* | *Very serious^a^* | | *Strong association* | | | *26* | *65* | *-* | *MD 4.1 higher (from 0.6 higher to 7.5 higher)* | | *⨁⨁⨁◯*  *Low* | |

a) data derived from a single study including few subjects.

PICO 8

| **Certainty assessment** | | | | | | | **Summary of findings** | | | | |
| --- | --- | --- | --- | --- | --- | --- | --- | --- | --- | --- | --- |
| **Participants (studies) follow up** | **Risk of bias** | **Inconsistency** | **Indirectness** | **Imprecision** | **Publication bias** | **Overall certainty of evidence** | **Study event rates (%)** | | **Relative effect (95% CI)** | **Anticipated absolute effect** | |
|  |  |  |  |  |  |  | **With comparison** | **With intervention** |  | **Risk with comparison** | **Risk difference between comparison and intervention** |
| **Total body weight loss (%) – All theraphies (vs placebo/no theraphy/lifestyle)** | | | | | | | | | | | |
| 21251 (13 RCT) | serious | serious | not serious | not serious | strong association | ⨁⨁⨁◯ Moderate | 10423 | 10828 | - | - | MD 7.53 higher (from 4.97 higher to 10.09 higher) |

PICO 9

| **Certainty assessment** | | | | | | | **Summary of findings** | | | | |
| --- | --- | --- | --- | --- | --- | --- | --- | --- | --- | --- | --- |
| **Participants (studies) Follow up** | **Risk of bias** | **Inconsistency** | **Indirectness** | **Imprecision** | **Publication bias** | **Overall certainty of evidence** | **Study event rates (%)** | | **Relative effect  (95% CI)** | **Anticipated absolute effect** | |
|  |  |  |  |  |  |  | **With comparison** | **With intervention** |  | **Risk with comparison** | **Risk difference between comparison and intervention** |
| **Total body weight loss (%) – All strategies (NMA)** | | | | | | | | | | | |
| 31,110 (64 RCT) | serious | serious | Not serious | Not serious | Strong association | ⨁⨁⨁◯ Moderate | 13285 | 17825 | - | - | MD **7.75** higher (from 6.38 higher to 9.11 higher) |

**CI:** Confidence interval; **MD:** Mean difference; **OR:** Odds ratio;

PICO 10

| **Certainty assessment** | | | | | | | **Sintesi dei risultati** | | | | |
| --- | --- | --- | --- | --- | --- | --- | --- | --- | --- | --- | --- |
| **Parteicipants (studies) Follow up** | **Risk of bias** | **Inconsistency** | **Indirectness** | **Imprecision** | **Publication bias** | **Overall certainty of evidence** | **Study event rates (%)** | | **Relative effect (95% CI)** | **Absoulte anticipated effect** | |
|  |  |  |  |  |  |  | **With comparison** | **With intervention** |  | **Risk with PBO** | **Difference with BMI 30-35** |
| **Total body weight loss (%) – All strategies (NMA)** | | | | | | | | | | | |
| 754 (5 RCT) | serious | serious | Not serious | Not serious | Strrong association | ⨁⨁⨁◯ Moderate | 275 | 479 | - | - | MD **18.37** higher (from 13.23 higher to 23.5 higher) |

**CI:** Confidence interval; **MD:** Mean difference; **OR:** Odds ratio;

a. Randomization and allocation often inadequately reported. b. Small sample size. c. High heterogeneity (I^2^> 50%). d. Data derived from a single study. e. Open label studies.

PICO 11

| **Certainty assessment** | | | | | | | **№ of patients** | | **Effect** | | **Certainty** | **Importance** |
| --- | --- | --- | --- | --- | --- | --- | --- | --- | --- | --- | --- | --- |
| **№ of studies** | **Study design** | **Risk of bias** | **Inconsistency** | **Indirectness** | **Imprecision** | **Other considerations** | **Weight loss** | **control** | **Relative (95% CI)** | **Absolute (95% CI)** |  |  |
| **Depression** | | | | | | | | | | | | |
| 16 | randomised trials | serious | not serious | not serious | not serious | none | 99/16405 (0.6%) | 88/12619 (0.7%) | **OR 0.72** (0.54 to 0.97) | **2 fewer per 1.000** (from 3 fewer to 0 fewer) | ⨁⨁⨁◯ Moderate |  |

**CI:** confidence interval; **MD:** mean difference; **OR:** odds ratio;

a. Small number of events

b. Significant Heterogeneityc. Funnel plot does not rule out publication bias
